# Preparation and epitope analysis of monoclonal antibodies against African swine fever virus DP96R protein

**DOI:** 10.1186/s12917-024-04043-6

**Published:** 2024-05-11

**Authors:** Chao Li, Xuan-ying Si, Xiao-ge Wang, Zhi-wei Yan, Hao-yu Hou, Long-qi You, Yin-long Chen, Ang-ke Zhang, Na Wang, Ai-jun Sun, Yong-kun Du, Gai-ping Zhang

**Affiliations:** 1 College of Animal Medicine, Henan Agricultural University, Zhengzhou, 450046 China; 2 National and International Joint Research Center for Animal Immunology, College of Animal Medicine, Henan Agricultural University, Zhengzhou, 450046 China; 3 Henan Engineering Laboratory of Animal Biological Products, Zhengzhou, 450046 China; 4 Longhu Advanced Immunization Laboratory, Zhengzhou, 450046 China

**Keywords:** African swine fever virus, DP96R protein, Monoclonal antibodies, Epitope analysis

## Abstract

**Background:**

Many proteins of African swine fever virus (ASFV, such as p72, p54, p30, CD2v, K205R) have been successfully expressed and characterized. However, there are few reports on the DP96R protein of ASFV, which is the virulence protein of ASFV and plays an important role in the process of host infection and invasion of ASFV.

**Results:**

Firstly, the prokaryotic expression vector of DP96R gene was constructed, the prokaryotic system was used to induce the expression of DP96R protein, and monoclonal antibody was prepared by immunizing mice. Four monoclonal cells of DP96R protein were obtained by three ELISA screening and two sub-cloning; the titer of ascites antibody was up to 1:500,000, and the monoclonal antibody could specifically recognize DP96R protein. Finally, the subtypes of the four strains of monoclonal antibodies were identified and the minimum epitopes recognized by them were determined.

**Conclusion:**

Monoclonal antibody against ASFV DP96R protein was successfully prepared and identified, which lays a foundation for further exploration of the structure and function of DP96R protein and ASFV diagnostic technology.

**Supplementary Information:**

The online version contains supplementary material available at 10.1186/s12917-024-04043-6.

## Background

African swine fever (ASF) is an acute and highly contagious infectious disease caused by African swine fever virus (ASFV) [1]. ASFV is an encapsulated virus containing a linear double-stranded DNA genome with a length of 170–190 kb. The DNA sequence has 150–170 ORFs, encoding more than 50 structural proteins and more than 100 non-structural proteins. The genome structure includes a central conserved region of about 125 kb, a left variable region of 38–47 kb and a right variable region of 13–16 kb [2]. ASFV is the only member of the ASFV family and the only known DNA arbovirus. Soft ticks of the genus, *Ornithodoros*, are the host and biological transmission medium of ASFV. The virulent strain of ASFV infects domestic pigs, leading to a short-course illness with a mortality rate as high as 100% [3]. ASF was first reported in Africa in 1909 [4], and spread to Europe through sub-Saharan Africa in 1920. Since it was introduced into the Republic of Georgia in 2007, epidemic ASF has been reported in 16 countries outside Africa [5]. In August 2018, the first case of ASF was diagnosed in Shenyang, Liaoning Province, China [6, 7]. Subsequently, Mongolia, Vietnam, Japan, the Philippines, South Korea, and South Africa have reported similar outbreaks [8, 9]. Due to the complexity of the virus itself, genetic diversity, and unknown interaction mechanism with the host [10], there is still a lack of specific treatment methods and effective commercial vaccines, which leads to the fact that all pigs infected with the virus can only be killed, causing huge losses to the domestic pig industry. At present, many ASFV proteins, such as p72, p54, p30, CD2v and K205R, have been expressed and identified by Chinese scholars, and the functions of some proteins have been studied. However, there are few reports about African swine fever virus DP96R protein at home and abroad. It is only known that this protein is the virulence protein of ASFV and plays an important role in the process of virus infection. Studies have shown that the virulence of ASFV E70 with deletion of DP96R gene and ASFV Georgia 2007/1 with deletion of both 9GL and DP96R gene decreased significantly [11]. DP96R protein can be expressed in the early stage of ASFV infection, and it can be detected in macrophages 2 h after infection with ASFV [12]. The study of DP96R can enrich people’s understanding of ASFV, therefore, this study intends to express DP96R protein, prepare and identify its specific monoclonal antibody, and analyze its recognized epitopes. The aim of this study is to provide an antibody tool for studying the function of ASFV DP96R protein and its role in the pathogenicity of ASFV infection, as well as for the research and development of ASFV diagnostic technology and vaccine.

## Materials and methods

### Cells, plasmids, experimental animals, and reagents

SP2/0 and Hela cells were obtained from BasalMedia (Shanghai, China), the pET-28a(+) and pCMV-N-Flag-DP96R plasmids were kindly provided by Professor Yong-kun Du of National and International Joint Research Center for Animal Immunology, Zhengzhou, China. The plasmid containing the DP96R gene sequence was synthesized by GenScript (Nanjing, China). SPF female BALB/c mice aged 6–8 weeks and aged 10–12 weeks were purchased from the Experimental Animal Center of Zhengzhou University. PEG1500, 50×HAT, 50×HT, kanamycin, Freund’s complete adjuvant and Freund’s incomplete adjuvant were purchased from Sigma (USA). The restriction endonucleases, BamH I and Xhol I, and the T4 DNA ligase were from NEB (USA). Ni-NTA agarose was from Qiagen (Germany) and IPTG was from Solaibao Biotech (Beijing, China). FITC-conjugated goat anti-mouse IgG was from Beyotime (Shanghai, China), HRP-conjugated goat anti-mouse IgG was from Proteintech (USA), and anti-His mouse monoclonal antibody was from TransGen Biotech (Beijing, China). The SDS-PAGE kit was from Epizyme Biotech (Shanghai, China) and the high-sensitivity ECL chemiluminescence kit was from Biodragon Biotech (Beijing, China). Fetal bovine serum and trypsin were from Gibco (USA). The mouse monoclonal antibody isotyping ELISA kit was from Proteintech Group (China). The anti-Flag magnetic agarose beads were from Biolinkedin (Shanghai, China).

### Primer design and PCR amplification of target gene fragments

Based on the DP96R gene sequence of the ASFV strain (GenBank accession number, MK128995.1), primers containing the restriction sites were designed by using BioXM. The primers were synthesized by Sangon Biotech (Shanghai, China). The sequence of DP96R-F is:5’-CG*GGATTC*ATGTCTACACATGATTGTTCTCTA-3’ (the underlined part is the BamH I restriction site), and the sequence of DP96R-R is:5’- CC*CTCGAG*ATTATTCTTCTGGATGGAGCGCAT-3’ (the underlined part is the Xhol I restriction site). The plasmid containing the DP96R gene was used as a template for PCR amplification. The PCR reaction contained the following: 1 µL template, 0.5 µL of upstream and downstream primers, 25 µL of PrimeSTAR ®Max enzyme and DEPC water added to make a final volume of 50 µL. The PCR conditions were: pre-denatured at 98 ℃ for 5 min, denatured at 98 ℃ for 30 s, annealed at 55 ℃ for 30 s for 35 cycles, extended at 72 ℃ for 10 min and then held at 4 ℃. The amplified products were separated by 1% agarose gel electrophoresis and the target fragments were recovered.

### Construction and verification of the recombinant expression vector

Using BamH I and Xho I, the gel recovery product containing the target gene was digested along with the pET-28a(+) vector for 2 h at 37 ℃. The corresponding fragments were recovered and ligated in a reaction containing 1 µl of 10×T4 DNA buffer, 0.5 µL of T4 DNA ligase, 6.5 µL of target DNA fragment, and 2 µL of vector fragment at 16 ℃ overnight. The ligation product was transformed into competent cells of *E. coli* BL21(DE3) on agar containing 50 µg/L kanamycin and cultured at 37 ℃ for 12 h. An isolated colony was selected and cultured in LB medium containing kanamycin on a shaker (220 rpm) at 37 ℃. The transformant culture was sent to Sangon Biotech (Shanghai, China) for sequencing and the correctly identified recombinant expression plasmid was named pET-28a (+) -DP96R.

### Induced expression, purification and identification of recombinant DP96R protein

The bacteria containing the prokaryotic expression vector pET-28a(+)-DP96R were inoculated at 1:100 in 200 mL of LB containing 50 µg/L kanamycin. When the OD_600_ reached 0.4, IPTG was added to a final concentration of 0.1 mM to induce DP96R expression. Induced cultures were incubated for 6 h in a shaker at 37 ℃ (220 rpm), then centrifuged at 8,000 g for 10 min. Bacterial pellets were resuspended in PBS, and disrupted with an ultrasonic probe for 40 min at 3 mW and 50% duty cycle on ice. Sonicates were centrifuged at 12,000 g for 10 min, and the supernatants were precipitated, and aliquots were separated by SDS-PAGE and stained. For purification, the remaining sample was loaded onto a nickel column and allowed to bind for 10 min. The column was washed with 10 mM NaCl, 100 mM Na_2_HPO_4_, 20 mM imidazole to remove impurities, then the target protein was eluted with 10 mM NaCl, 100 mM Na_2_HPO_4_, and 250 mM imidazole. This method yielded high purity DP96R protein as determined by SDS-PAGE/western immunoblot, which was aliquotted and stored at -80 ℃.

### Preparation of monoclonal antibody against DP96R recombinant protein

The purified DP96R recombinant protein was mixed with Freund’s adjuvant in equal volumes, emulsified, and each mouse aged 6–8 weeks was intraperitoneally immunized with 25 µg. After the first immunization with DP96R in Freund’s complete adjuvant, the mice were immunized twice every two weeks with Freund’s incomplete adjuvant, for a total of three times. Blood was collected from the tip of the tail one week after the third immunization and the serum antibody titer was measured by indirect ELISA. The mouse with the highest titer was selected for booster immunization which meant the mouse was injected with 100 µg protein intraperitoneally. After 3 days of booster immunization, the mouse was euthanized and spleen was removed. Splenocyte suspensions were prepared and fused with SP2/0 cells by PEG1500 treatment at a ratio of 10:1. The fused cells were subcloned by limiting dilution, and screened by indirect ELISA to select DP96R protein-specific hybridoma cell lines. The selected cell lines were expanded, frozen, and stored in liquid N_2_.

### Preparation of ascites and measurement of antibody titer

Female BALB/c mice aged 10–12 weeks were injected intraperitoneally with 200 µL of Freund’s incomplete adjuvant, and after 7 days, each mouse was injected with 1 × 10^6^ hybridoma cells. After the abdominal cavity of the mice swelled, the ascites fluid was extracted with a syringe, centrifuged at 12,000 g for 10 min, aliquotted in cryotubes and stored at -80 ℃. The DP96R antibody titer was determined by indirect ELISA as follows. Purified DP96R protein (100 µl of 5 µg/mL) was added to each well of a 96-well enzyme-labeled microtiter plate and incubated at 4 ℃ for 12 h. The plate was washed three times with PBST, blocked with 5% skimmed milk at 37 ℃ for 2 h, and then washed three times again with PBST. The antibody-containing ascites was added at dilutions of 1:1,000, 1:10,000, 1: 100,000, 1: 500,000, 1: 2,500,000, 1:12,500,000 and the plates were incubated at 37 ℃ for 1 h. After the plates were washed three times, HRP-conjugated goat anti-mouse IgG was added (100 µL/well) at a dilution of 1:2000, and incubated at 37 ℃ for 1 h. After three times washing, tetramethylbenzidine substrate was added and incubated 15 min in the dark. The reaction was terminated by addition of 3 M H_2_SO_4_, and the absorbance at 450 nm was measured. An A_450_ ratio of sample (S) / negative control (N) ≥ 2.1 was judged to be positive. The highest dilution yielding a positive ELISA signal was considered the antibody titer.

### Identification of the monoclonal antibodies

Purified DP96R protein was separated by SDS-PAGE and His-tagged ASFV p30 protein (previously prepared in our lab) was used as the control. The proteins were transferred to a PVDF membrane (300 mA, 75 min) and blocked with TBST containing 5% skim milk at room temperature for 2 h. Hybridoma ascites which was diluted with 5% skim milk (1:500) and anti-His mouse mAb which was diluted with 5% skim milk (1:2000) were added as primary antibodies. After incubation at 37 ℃ for 1 h, the membrane was washed three times with TBST (10 min each) and HRP-conjugated goat anti-mouse IgG diluted with 5% skim milk(1:5000) was added as secondary antibody at 37 ℃ for 1 h. Lastly, the membrane was washed three times with TBST (10 min each), and signal intensity was determined by enhanced chemiluminescence using an Amersham Imager680.

The monoclonal antibody subtype was identified using a commercial mouse monoclonal antibody isotyping ELISA kit.

Hela cells were incubated at 37 ℃ in a 24-well plate to a density of about 60%, then transfected with the eukaryotic expression plasmid pCMV-N-Flag-DP96R at a dose of 1 µg/well; Hela cells transfected with pCMV-N-Flag vector served as control. After 24 h, the cells were fixed with 4% paraformaldehyde for 30 min at room temperature, and the cells were blocked with 1% BSA after permeabilization. Hybridoma ascites (1:500) was added as primary antibody, FITC-conjugated goat anti-mouse IgG (1:500) was used as secondary antibody. After staining the nucleus with DAPI, the results were observed under a fluorescence microscope.

The pCMV-N-Flag-DP96R plasmid was transfected into Hela cells and the cells were incubated at 37 ℃. After 24 h, cell samples were collected, and the supernatant was collected after cell lysis. One supernatant sample was boiled for 5 min and temporarily stored at 4 ℃ as the Input experimental group, while another sample was incubated overnight with anti-Flag agarose beads. The overnight incubated sample was then centrifuged and boiled as the IP experimental group for electrophoresis. After electrophoresis, the protein samples were transferred to the PVDF membrane. After blocking and incubation with the corresponding antibody, the results was determined by enhanced chemiluminescence using an Amersham Imager680.

### Identification of DP96R antigenic epitopes

The secondary structure of a protein has a large influence on the epitope. The chemical bond energies of alpha-helices and beta-sheets are relatively high, and these can firmly maintain the structure of a protein. Such an epitope can be difficult to chimerize with antibodies as it is often located inside the protein, so it is unlikely to be the region where the antigenic epitopes are located. In contrast, the flexibility of β-turns and random coils in a protein creates a loose structure that is relatively easy to twist and display on the surface of the protein. Such a prominent structure is conducive to chimerize with antibodies and is more likely to serve as an antigenic epitope. Seven peptides (P1-P7) were designed and synthesized according to the amino acid sequence and the predicted secondary structure of the DP96R protein. A series of peptides was synthesized again with a one-amino acid step from both ends after the peptides recognized by monoclonal antibodies were preliminarily determined by Dot-blot and peptide-based ELISA. Subsequently, the precisely antigenic epitope recognized by the mAbs were further identified using Dot-blot and peptide-based ELISA. And the three-dimensional structure and location of the protein epitopes was predicted and displayed by PyMOL.

### Conservative analysis of DP96R antigenic epitopes

In order to evaluate the conservation of the two identified epitopes of ASFV DP96R protein in different ASFV strains, the amino acid sequence sets of 25 ASFV DP96R strains were randomly retrieved from GenBank database and analyzed by DNAMAN software.

### Treatment of laboratory animals

The experimental animals used in the experiment were euthanized by inhaling carbon dioxide. Put the experimental animals into the carbon dioxide anesthesia box, open the carbon dioxide valve, and wait until the animals gradually lose consciousness. Then increase the carbon dioxide concentration to 100%. The animals will appear unconscious and continue to ventilate for 2 min to determine the death of the animals.

## Results

### Construction and characterization of the prokaryotic recombinant plasmid of the DP96R gene

**Fig. 1:**
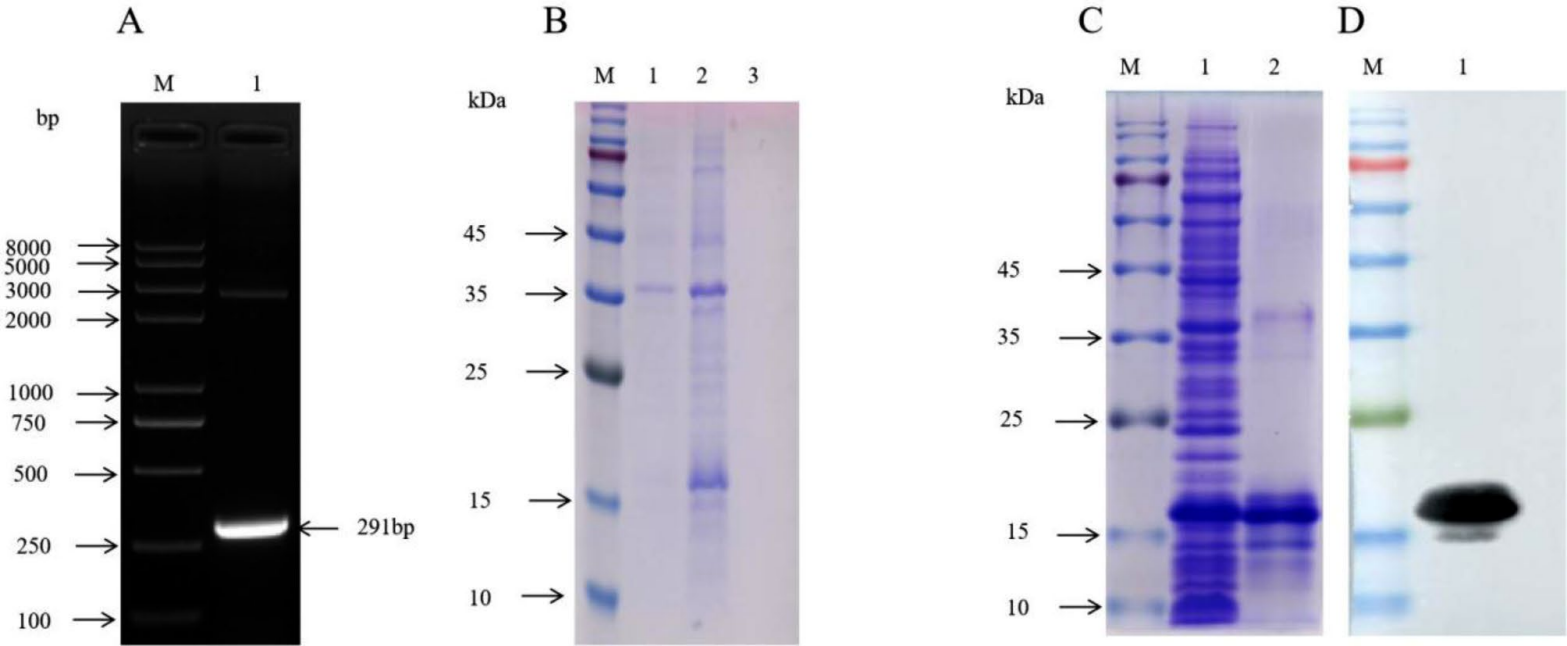
Identification of the PCR amplicon as the DP96R gene; expression, purification and analysis of the recombinant DP96R protein. (**A**) *M*, DNA marker; lane 1, PCR amplification products of the DP96R gene. (**B**) SDS-PAGE analysis of the prokaryotically expressed recombinant DP96R protein (~ 15 kDa). *M*, relative size marker standards for proteins; lane 1, non-induced bacterial culture; lane 2, supernatant after induction of bacterial culture; lane 3, precipitation of proteins from culture supernatants of induced bacteria. (**C**) SDS-PAGE analysis of purified recombinant DP96R protein. *M*, relative size marker standards for proteins; lane 1, non-purified sample; lane 2, purified recombinant DP96R protein. (**D**) Western blot analysis of purified recombinant DP96R protein. *M*, relative size marker standards for proteins; lane 1, purified recombinant DP96R protein

The plasmid containing the DP96R gene sequence was used as a template for PCR amplification, and the size of the PCR product as determined by electrophoresis on a 1% agarose gel was about 291 bp, which was consistent with the predicted DP96R fragment size (Fig. 1A). The target gene fragment was inserted into the pET-28a(+) vector, and the recombinant expression plasmid was verified by PCR and digest with restriction endonucleases, BamH I and Xho I. Sequence alignment showed that the expression plasmid contained the DP96R gene sequence, which proved that the correct gene sequence was inserted and the recombinant plasmid, pET-28a(+)-DP96R, was constructed correctly.

### Expression and purification of DP96R recombinant protein

The recombinant *E. coli* strain pET-28a-DP96R/BL21 was induced and expressed, and the non-induced strain was used as control. The strain was induced with 0.1 mM IPTG at 37 ℃ for 6 h with shaking at 220 rpm. Cultures were centrifuged, and precipitates of the supernatants from the pET-28a-DP96R/BL21 strain had an obvious band at 15 kDa, which was absent from the non-induced recombinant and the induced strain containing empty vector. This verified the successful cloning of the inducible expression of DP96R and the recombinant protein was mainly expressed in soluble form (Fig. 1B). After the expressed protein was purified by nickel column affinity chromatography, SDS-PAGE showed a strong target band at 15 kDa (Fig. 1C), which confirmed that purification of recombinant DP96R protein resulted in high purity. Western blotting with anti-His mouse mAb as primary antibody was used to detect DP96R protein with HRP-conjugated goat anti-mouse IgG as secondary antibody. A single band was detected at 15 kDa (Fig. 1D), showing that the recombinant protein was correctly expressed and purified.

### Preparation of monoclonal antibodies and determination of antibody titer

On the seventh day after fusion, the cells were screened by indirect ELISA and four monoclonal cell lines that stably secreted specific antibodies against DP96R protein were detected: 2F1D4C3, 6F1B2C5, 5C10D4C9, and 9C10F9D5. The mAbs prepared from these four hybridoma cells showed an effective titer of 1:500,000 (Table 1).

**Table 1:** Results of titer determination of mouse ascites with monoclonal antibody

Dilution	2F1D4C3		5C10D4C9		6F1B2C5		9C10F9D5		Negative 1: 1000
	OD_450 nm_	*P*/*N*	OD_450 nm_	*P*/*N*	OD_450 nm_	*P*/*N*	OD_450 nm_	*P*/*N*	
1:1000	2.3808	21.98	2.4652	22.76	2.3759	21.94	2.4478	22.60	0.1083
1:10000	2.3606	21.80	2.0511	18.94	2.2681	20.94	2.3286	21.50	
1:100000	1.8375	16.97	1.0526	9.72	1.9444	17.95	2.1649	19.99	
1:500000	0.9388	8.67	0.462	4.27	1.1274	10.41	1.784	16.47	
1:2500000	0.3523	3.25	0.2948	2.72	0.389	3.59	0.9883	9.13	
1:12500000	0.1413	1.30	0.2688	2.48	0.1472	1.36	0.3461	3.20	

### Western blot detection and subtype identification

**Fig. 2:**
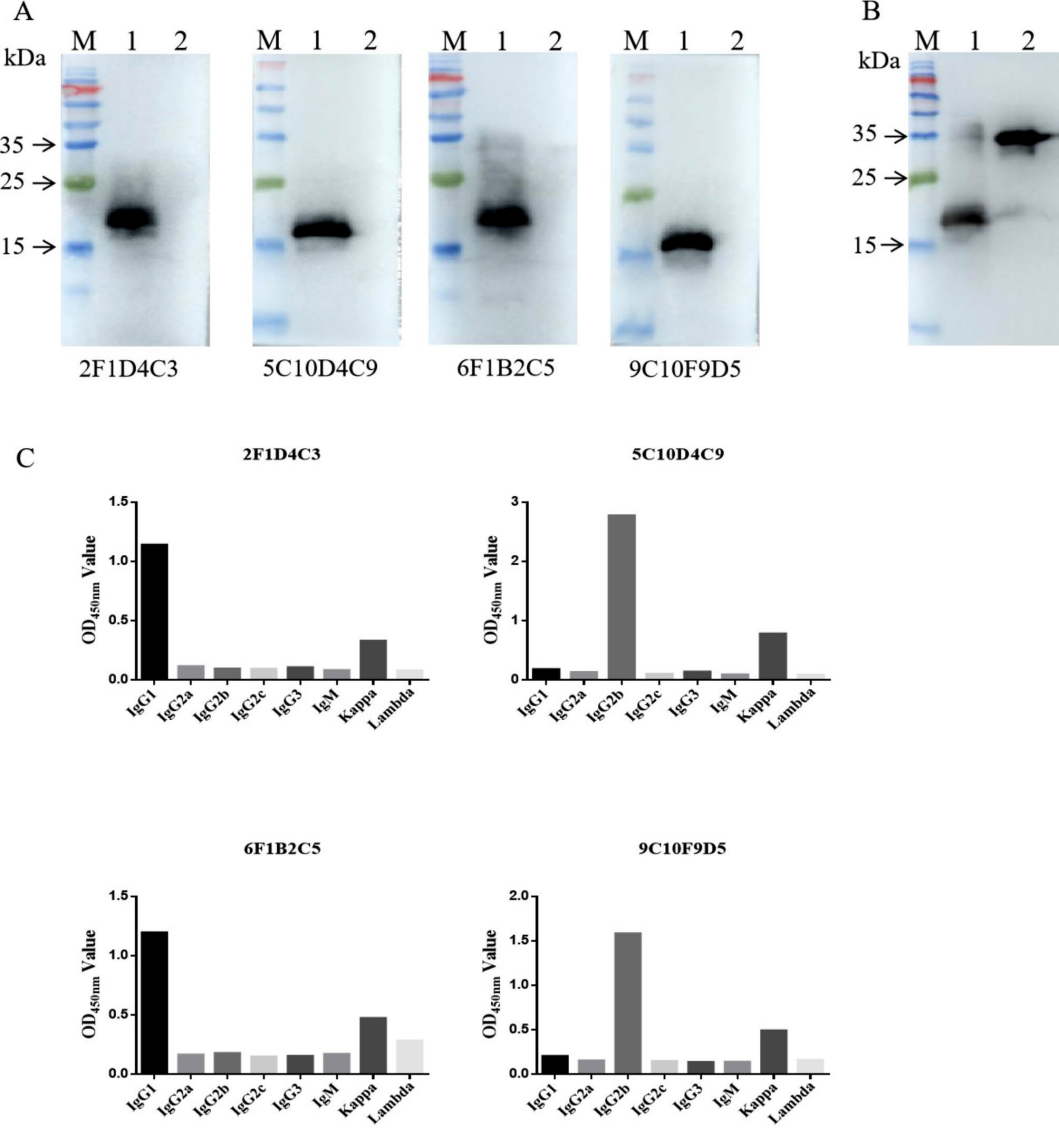
Western blot detection and subtype identification. (**A & B**)Western blotting. *M*, relative size marker standards for proteins; lane 1, purified recombinant DP96R protein; lane 2, purified recombinant P30 protein. (**C**) Subtype identification. The mAb subtype was identified using a commercial mouse mAb isotyping ELISA kit

Purified DP96R recombinant protein was run on SDS-PAGE, transferred to a PVDF membrane, and the p30 protein with His tag was used for detection. The prepared mouse mAbs and the anti-His mouse mAb were used as primary antibodies for western blot analysis, and the results showed a single band at 15 kDa (Fig. 2A), which was consistent with the expected size and did not react with p30 protein (Fig. 2B). Thus, the mAbs specifically recognized DP96R protein with good specificity and reactivity.

The subtypes of the mAbs were identified using a commercial kit, and the results showed that both the heavy chain isoforms of 2F1D4C3 and 6F1B2C5 were IgG1 and the light chain isoforms were kappa. The heavy chain isoforms of 5C10D4C9 and 9C10F9D5 strains were IgG2b and the light chain isoforms were all kappa (Fig. 2C).

### Identification of monoclonal antibody with IFA

**Fig. 3:**
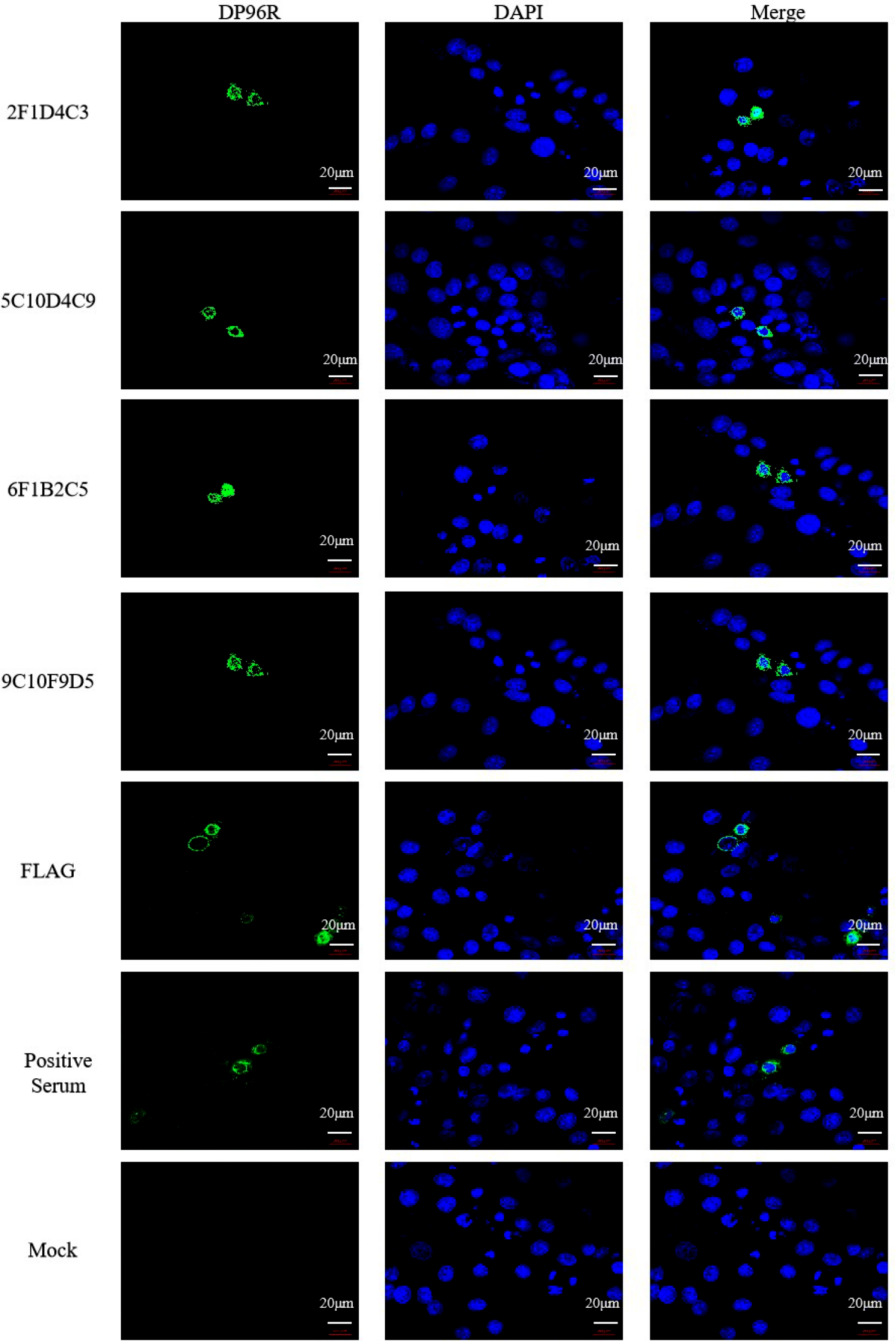
IFA of mAb recognition of DP96R expressed by eukaryotic cells. A DP96R expression vector was cloned into Hela cells and the protein was overexpressed. The prepared mAb was used as the primary antibody, and FITC-conjugated goat anti-mouse IgG was used as secondary antibody for the fluorescence assay

The pCMV-N-Flag-DP96R eukaryotic expression plasmid (with Flag tag) was transfected into Hela cells and then detected by immunofluorescence assay (IFA).The results showed that the prepared DP96R mAbs specifically interacted with Hela cells that overexpressed DP96R protein (Fig. 3), verifying that our mAbs were able to recognize DP96R protein expressed by eukaryotic cells.

### Identification of monoclonal antibody with co-IP

**Fig. 4:**
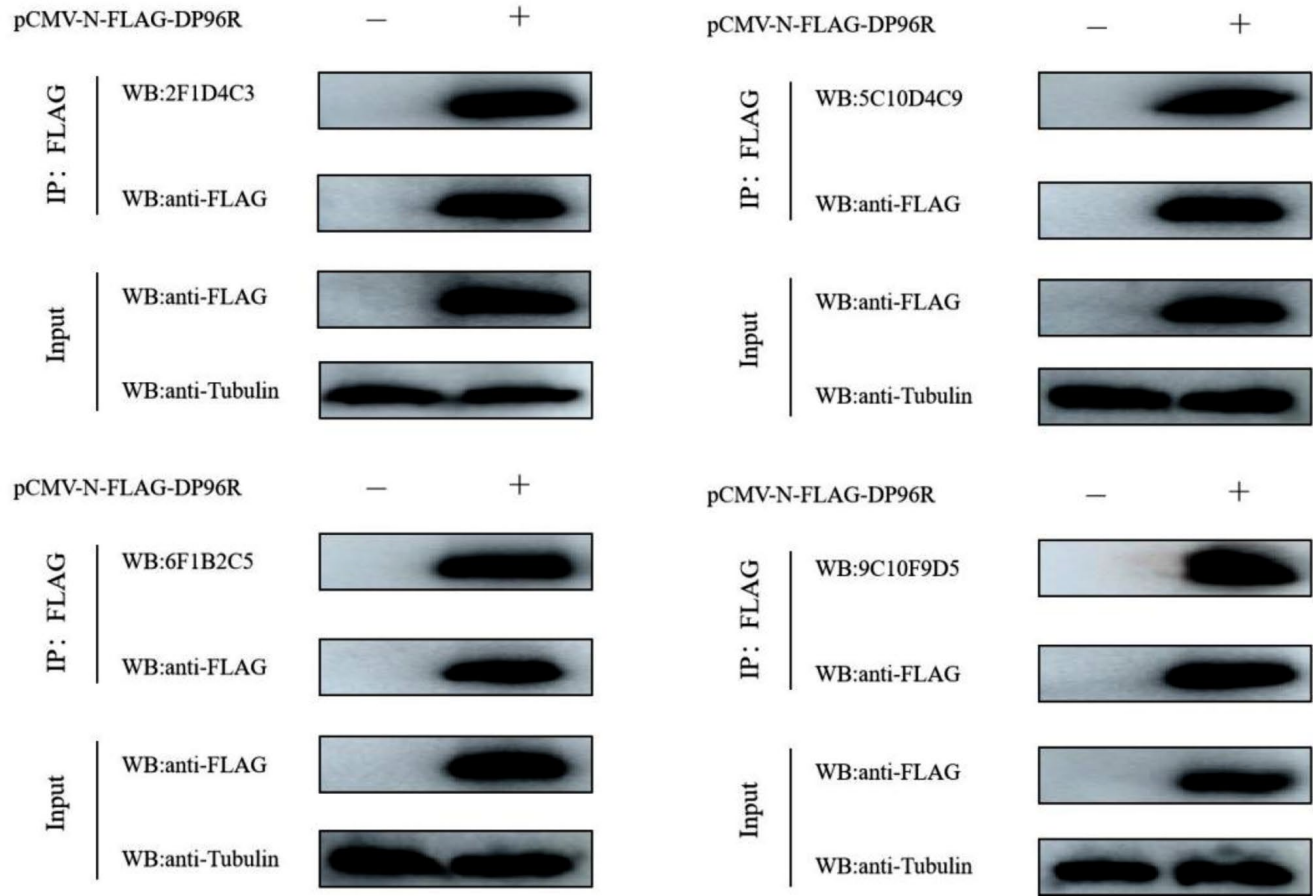
Co-IP identification of DP96R protein. The eukaryotic expression plasmid for Flag-tagged DP96R was transfected into Hela cells, and the empty vector plasmid was used as the control. Samples were collected for protein assays after transfection for 24 h

Co-IP results showed that as the Input sample, Hela cells transfected with ++*pCMV-N-Flag-DP96R *reacted with mAb (Fig. 4), but there was no specific band in Hela cells transfected with the empty pCMV-N-FLAG vector. With immunoprecipitated samples, the Flag-tagged DP96R protein conjugate expressed by Hela cells transfected with pCMV-N-Flag-DP96R could be recognized by the mAb, but there was no specific band in Hela cells transfected with the empty pCMV-N-Flag vector. After the Flag-tagged DP96R protein bound to anti-Flag agarose, the prepared mAb recognized the DP96R protein bound to the beads. This shows that the DP96R mAbs can be used in western blot assays as the primary antibodies.

### Identification of DP96R antigenic epitopes

**Fig. 5:**
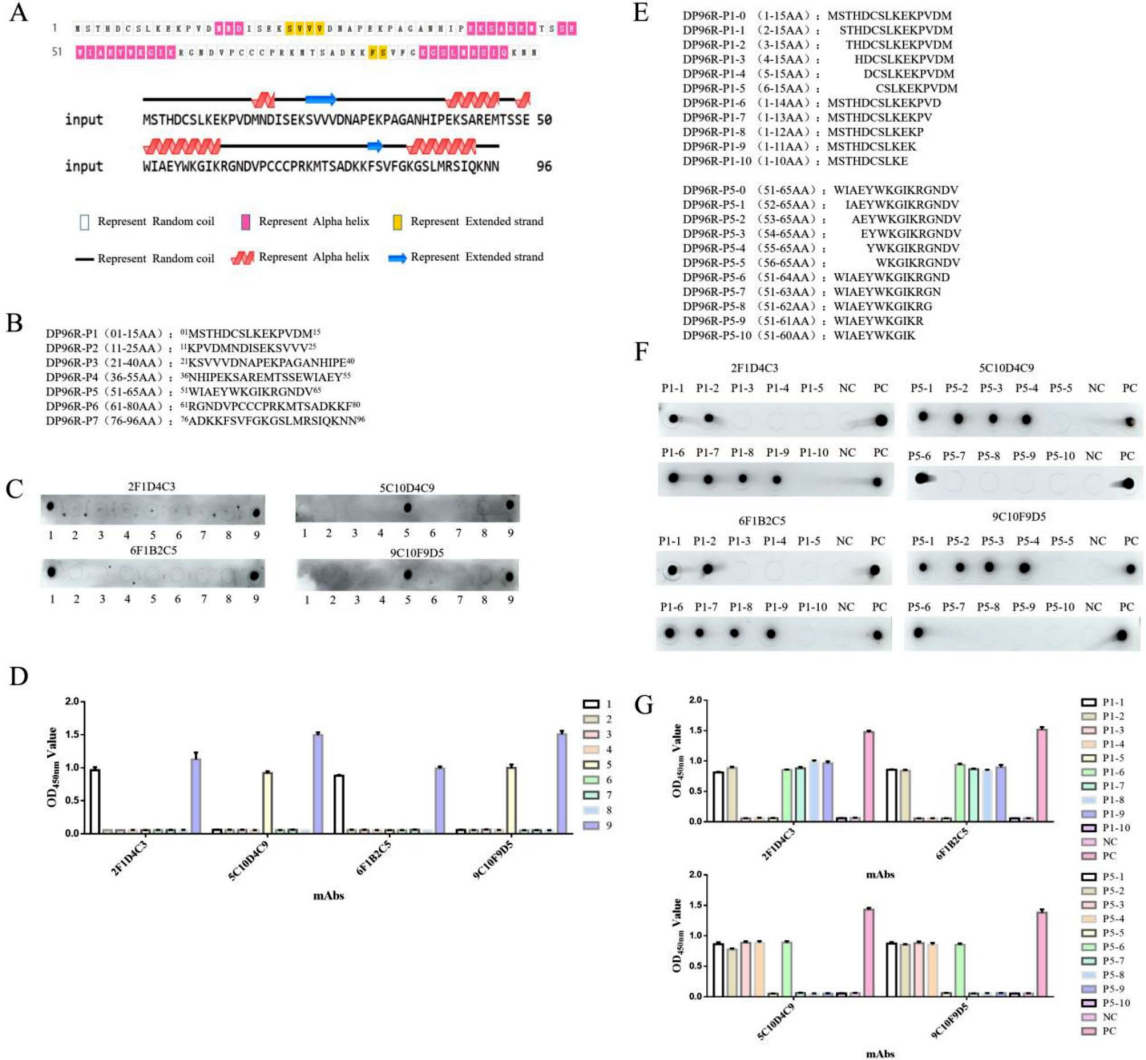
Identification of antigenic epitopes. (**A**) Prediction of secondary structure of DP96R protein. (**B**) Full-length DP96R protein was truncated into seven peptides. (**C & D**) Identification of truncated peptides by dot-blot and peptide-based ELISA. The lane numbers, 1–7 correspond to the peptides P1-P7; 8 is the negative control and 9 is the positive control; the error bars refer to standard deviation (SD) of three replicates from one experiment. (**E**) The peptide sequence was truncated stepwise from both ends. (**F & G**) Dot-blot and peptide-based ELISA identification of the further truncated peptides

In order to determine the antigenic epitopes of the DP96R protein, it was cleaved into seven segments for peptide analysis (Fig. 5B) according to the predicted secondary structure of the DP96R protein (Fig. 5A), and the truncated peptides were identified by dot-blot and peptide-based ELISA. The results showed that the mAbs of 2F1D4C3 and 6F1B2C5 recognized the first truncated peptide, and the epitopes were located in the ^01^MSTHDCSLKEKPVDM^15^ region. The antibodies of 5C10D4C9 and 9C10F9D5 bound to the fifth truncated peptide, in the ^51^WIAEYWKGIKRGNDV^65^ region (Fig. 5C). The results of peptide-based ELISA were consistent with those of dot-blot (Fig. 5D). In order to determine the minimum epitope for recognition by the four hybridomas screened, a series of peptides were synthesized by truncating an amino acid from both ends (Fig. 5E). The dot-blot and peptide-based ELISA results showed that the minimum epitope recognized by the mAbs of 2F1D4C3 and 6F1B2C5 was ^03^THDCSLKEK^11^. The minimum epitope recognized by the mAbs of 5C10D4C9 and 9C10F9D5 was ^55^YWKGIKRGND^64^ (Fig. 5F). And the results of peptide-based ELISA were consistent with those of dot-blot (Fig. 5G).

### Determination of how conserved are the DP96R antigenic epitopes

**Fig. 6:**
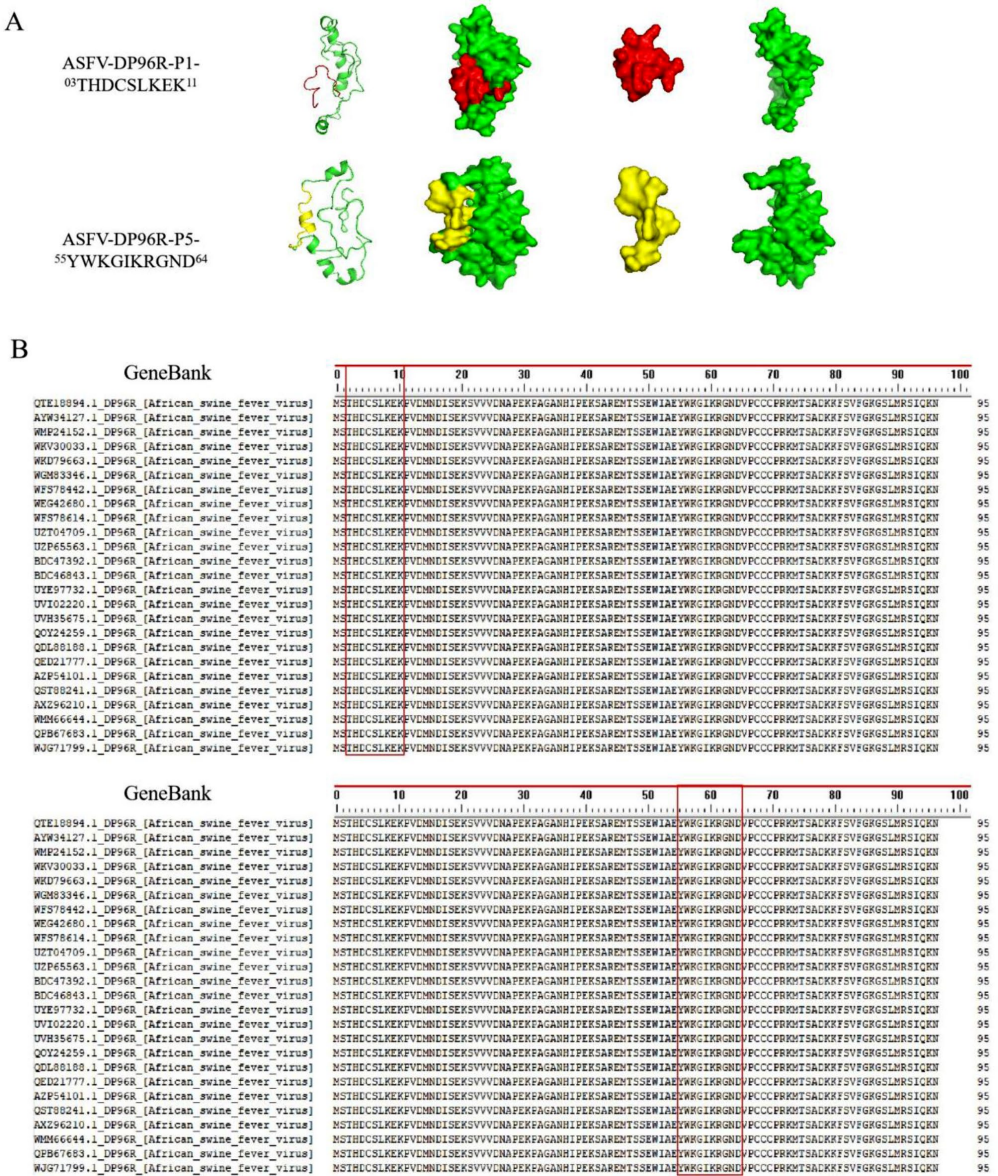
Depiction of antigenic epitopes and determination of how conserved they are. (**A**) PyMOL was used to compare the two identified epitopes and show the minimum epitope. The minimum epitope recognized by the mAbs of 2F1D4C3 and 6F1B2C5 is highlighted in red. The minimum epitope recognized by the mAbs of 5C10D4C9 and 9C10F9D5 is highlighted in yellow. (**B**) Comparison of the amino acid sequence of DP96R protein of different strains

PyMOL software was used to depict the antigenic epitopes (Fig. 6A). Comparison of the identified epitopes with the amino acid sequences of 25 random ASFV DP96R strains showed that the sequences of the two epitopes recognized by the mAb were highly conserved (Fig. 6B).

## Discussion

As a member of the African Swine fever virus family, ASFV has swept through more than 50 countries and regions around the world, including China, since 1921 [13, 14]. ASFV is a virus that that causes a highly lethal infection in domestic pigs and wild boars. Since it was introduced into the Caucasus in 2007, it has continued to spread in Eastern Europe and Russia. At present, it has spread to many countries and regions such as western Europe, China and Southeast Asia, posing a serious threat to the global hog-farming industry [15, 16]. With the continuous development of commodity trade, the transmission distance and spread range of the disease have gradually expanded. The existence of long-term vectors such as soft ticks [17, 18], have further increased the difficulty of disease control [19, 20]. Various vaccine strategies for ASF have been tested. Previously, the antigen-based method was focused on inducing a neutralizing serological reaction. However, the use of CD2v as antigen did not result in sufficient immune protection. Since then, many subunit vaccine methods have utilized p54 and p30, and the data showed that simultaneous immunization with p54 and p30 could delay the appearance of clinical symptoms of ASF. Therefore, it is of great significance to study the function of ASFV proteins and their functional groups in depth for rational, targeted design of vaccines [21–23]. The preparation of monoclonal antibodies (mAbs) against ASFV protein is necessary for the study of the gene function of the virus, and also provides materials for ASFV detection and vaccine research. Therefore, the preparation of mAbs against ASFV protein has become a hot research topic.

The protein composition of ASFV virions is complex, and the function and antigenic characteristics of viral protein are still unclear. P72 is a late-stage expressed protein, accounting for about 30% of the total viral protein, and its immunogenicity is strong and stable [24]. P54 is an early transmembrane protein, a key element in the formation of the viral envelope, and participates in the invasion of virus into cells. P54 mediates the transport of ASFV into cells, induces apoptosis in infected cells, and is considered to be one of the main virulence proteins [25]. P30 is an early protein that participates in virus invasion and interferes with host cell transcription and translation [26]. Up to the present, there have been many reports on research into the production of ASFV p30 mAbs, but there is little data related to the DP96R protein system.

Previous studies have shown that the protein encoded by the ASFV gene, DP96R, is a virulence protein of the African swine fever virus (ASFV) and the protein encoded by this gene is crucial for viral infectivity and pathogenicity. The DP96R gene sequence encoding the DP96R protein is highly conserved in different viral genome sequences. The DP96R protein can inhibit the phosphorylation of TBK1 and IRF3 induced by cGAS-STING, which is an important target for virus in immune escape and vaccine research [27]. In this study, we inducibly expressed DP96R protein in a prokaryotic expression system, used it to immunize mice, and prepared mAbs against the protein. We also identified its antigenic epitopes in order to lay a foundation for the development of serological diagnostics for ASFV and to study the biological function of DP96R protein.

## Conclusions

In this study, the prokaryotic expression vector of DP96R gene was successfully constructed, and the expression of DP96R protein was induced by E. coli prokaryotic expression system, and then the protein was purified by nickel column affinity chromatography. The purified DP96R protein was used to immunize mice. Four monoclonal cells of DP96R protein were successfully prepared by cell fusion and subclonal screening experiments, and the titer of the prepared ascites antibody was as high as 1:500 000. Subsequently, the prepared mAb was identified by Western blot, IFA and co-IP experiments, and the results showed that the prepared mAb had good specificity. The subtype of the mAb was subsequently identified, and finally the minimal antigen sequence recognized by the prepared mAb was determined by synthetic peptide method.

### Electronic supplementary material

Below is the link to the electronic supplementary material.

Supplementary Material 1

## Data Availability

All data supporting our findings is contained within the manuscript.
