# Correction: Preparation and epitope analysis of monoclonal antibodies against African swine fever virus DP96R protein

**DOI:** 10.1186/s12917-024-04165-x

**Published:** 2024-07-20

**Authors:** Chao Li, Xuan-ying Si, Xiao-ge Wang, Zhi-wei Yan, Hao-yu Hou, Long-qi You, Yin-long Chen, Ang-ke Zhang, Na Wang, Ai-jun Sun, Yong-kun Du, Gai-ping Zhang

**Affiliations:** 1 College of Animal Medicine, Henan Agricultural University, Zhengzhou, 450046 China; 2 National and International Joint Research Center for Animal Immunology, College of Animal Medicine, Henan Agricultural University, Zhengzhou, 450046 China; 3 Henan Engineering Laboratory of Animal Biological Products, Zhengzhou, 450046 China; 4 Longhu Advanced Immunization Laboratory, Zhengzhou, 450046 China

**Correction** **:** **BMC Vet Res 20, 191 (2024)**

**https://doi.org/10.1186/s12917-024-04043-6**

Following the publication of the original article [1], the authors identified an error in Figure 3. The correct and incorrect figures are given below.

Incorrect figure 3:

**Fig. 3:**
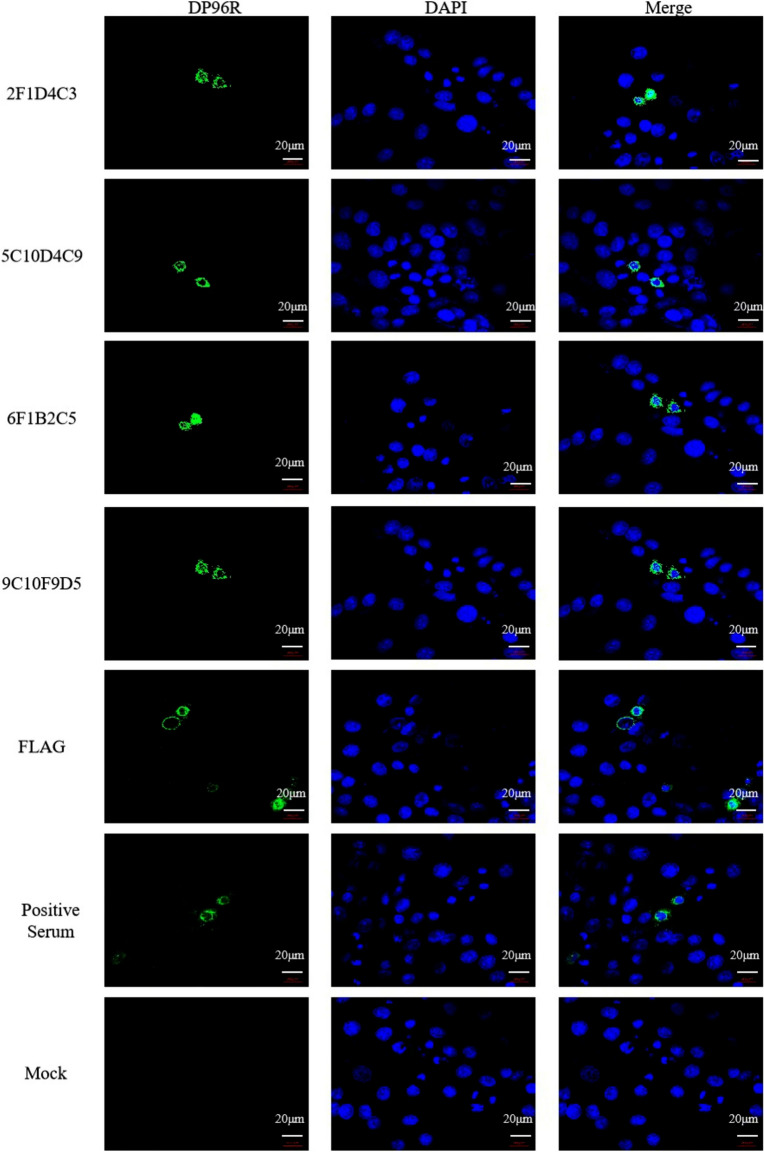
IFA of mAb recognition of DP96R expressed by eukaryotic cells. A DP96R expression vector was cloned into Hela cells and the protein was overexpressed. The prepared mAb was used as the primary antibody, and FITC-conjugated goat anti-mouse IgG was used as secondary antibody for the fluorescence assay

Correct figure 3:

**Fig. 3:**
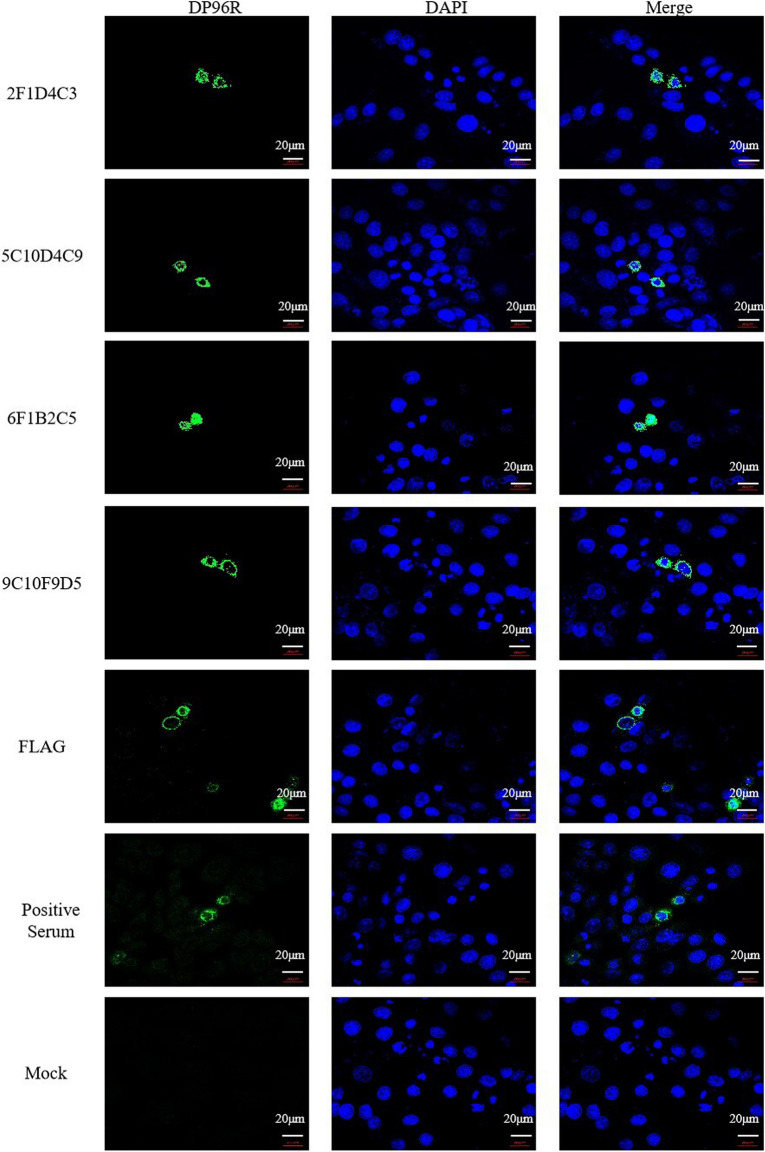
IFA of mAb recognition of DP96R expressed by eukaryotic cells. A DP96R expression vector was cloned into Hela cells and the protein was overexpressed. The prepared mAb was used as the primary antibody, and FITC-conjugated goat anti-mouse IgG was used as secondary antibody for the fluorescence assay
