# Supplementary material for: Preparation and epitope analysis of monoclonal antibodies against African swine fever virus DP96R protein
Source: BMC Vet Res. 2024 May 11;20:191. doi: 10.1186/s12917-024-04043-6 (PMC11088100; doi:10.1186/s12917-024-04043-6)

Fig.1

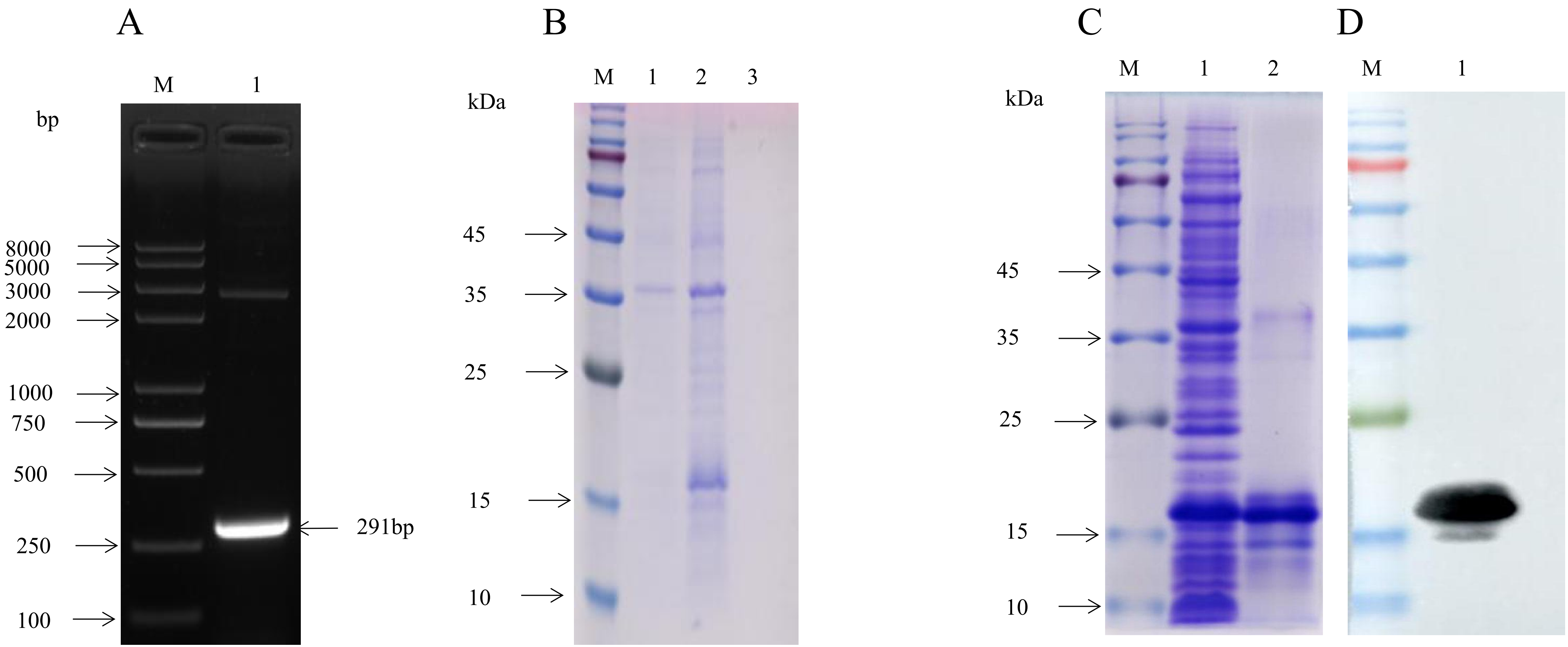
A
M
1
bp
8000
5000
3000
2000
1000
750
500
250
100
291bp
B
M
1
2
3
kDa
45
35
25
15
10
C
M
1
2
kDa
45
35
25
15
10
D
M
1

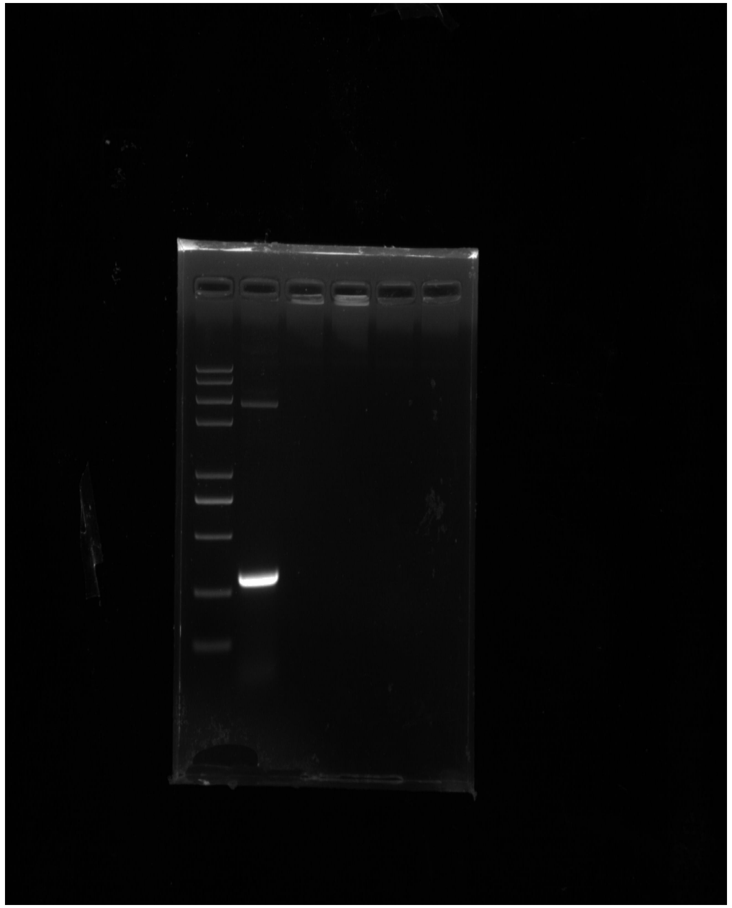
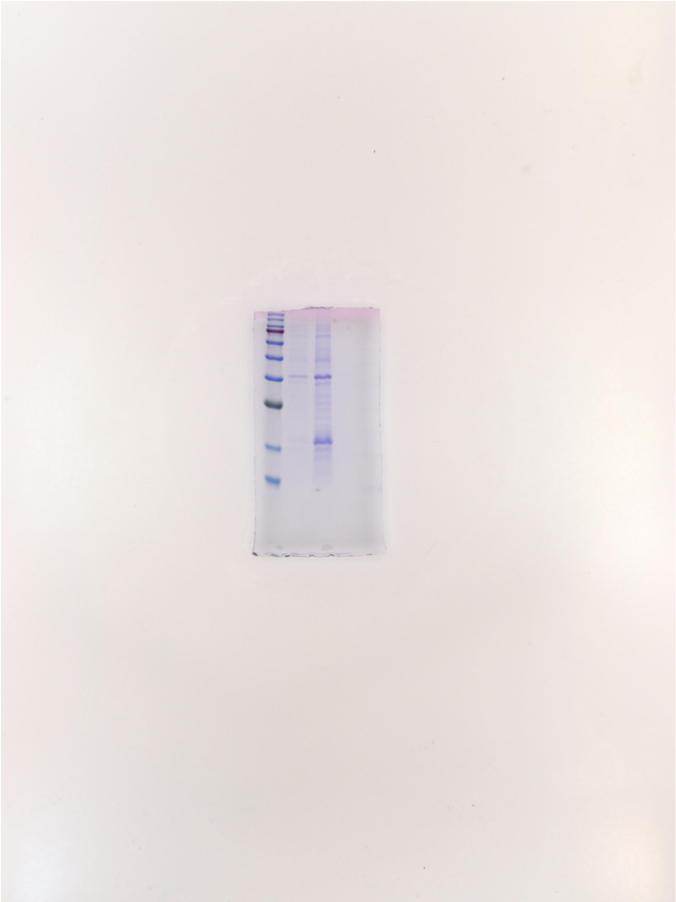
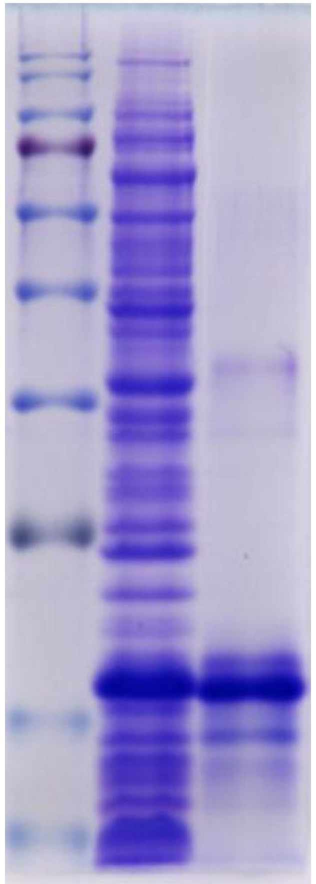
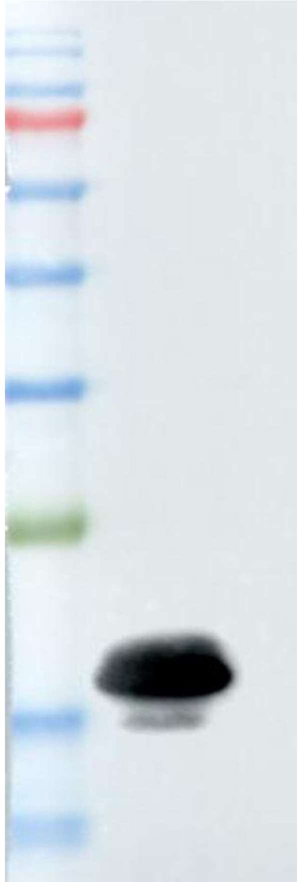

Fig.2

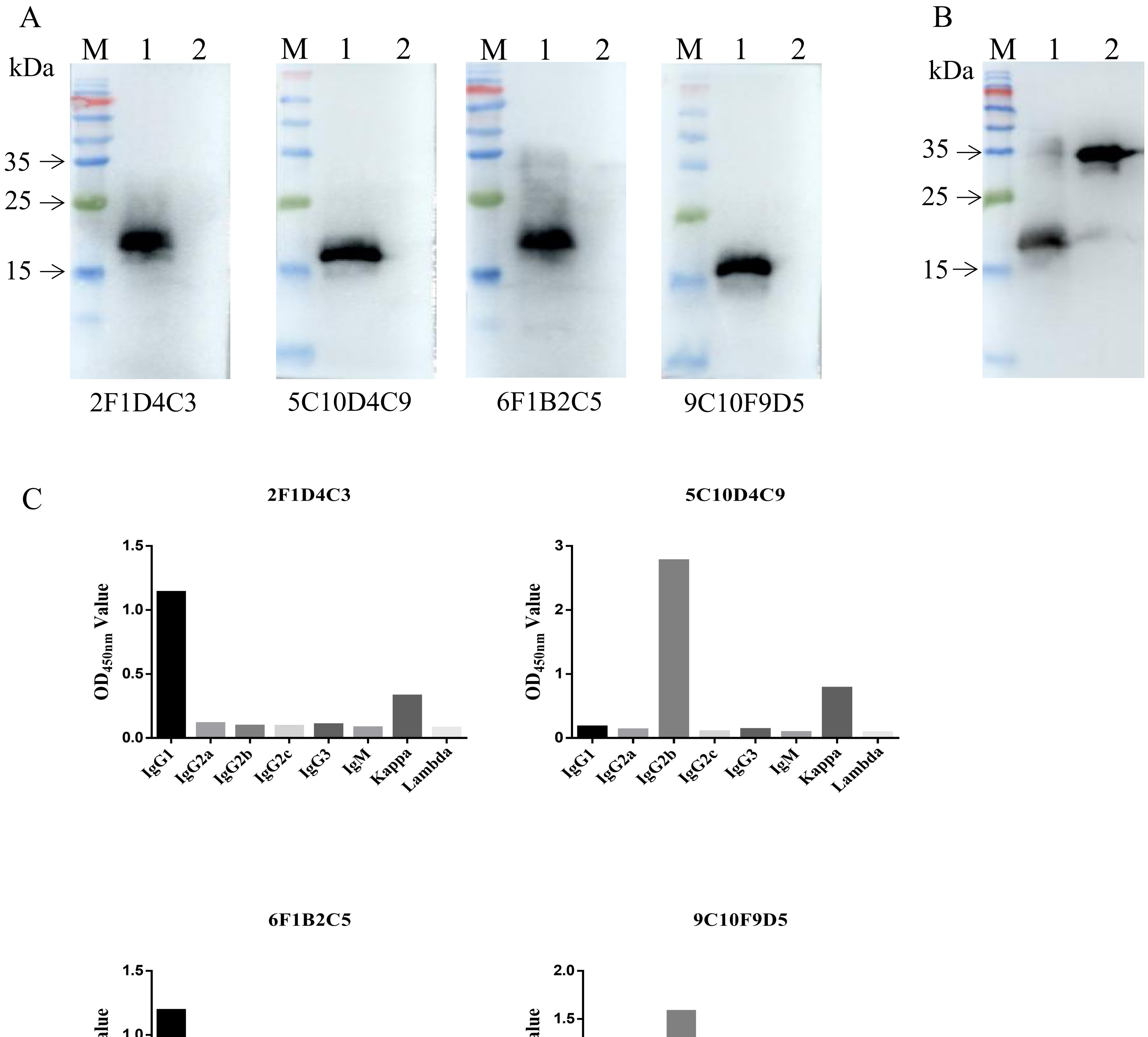
A
M 1 2
kDa
35
25
15
2F1D4C3
5C10D4C9
6F1B2C5
9C10F9D5
B
M 1 2
kDa
35
25
15
C
2F1D4C3
5C10D4C9
6F1B2C5
9C10F9D5
OD450nm Value
0.0
0.5
1.0
1.5
0
1
2
3
2.0
IgG1
IgG2a
IgG2b
IgG2c
IgG3
IgM
Kappa
Lambda

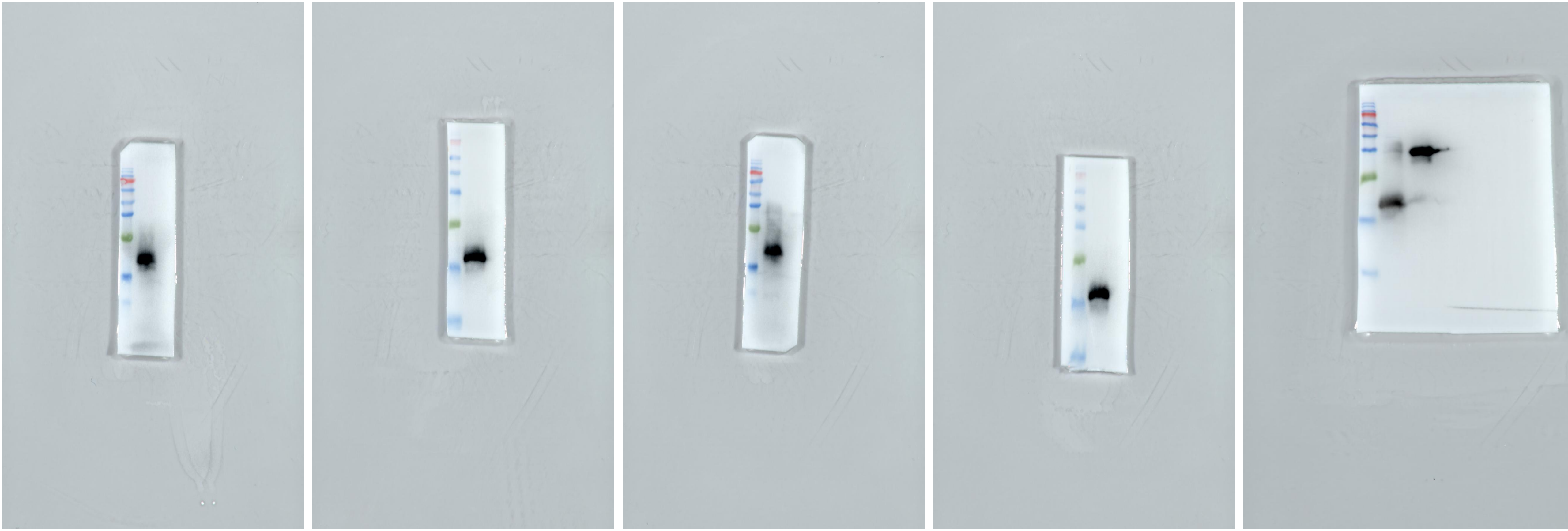

2F1D4C3 5C10D4C9 6F1B2C5 9C10F9D5 anti-His

Fig.4

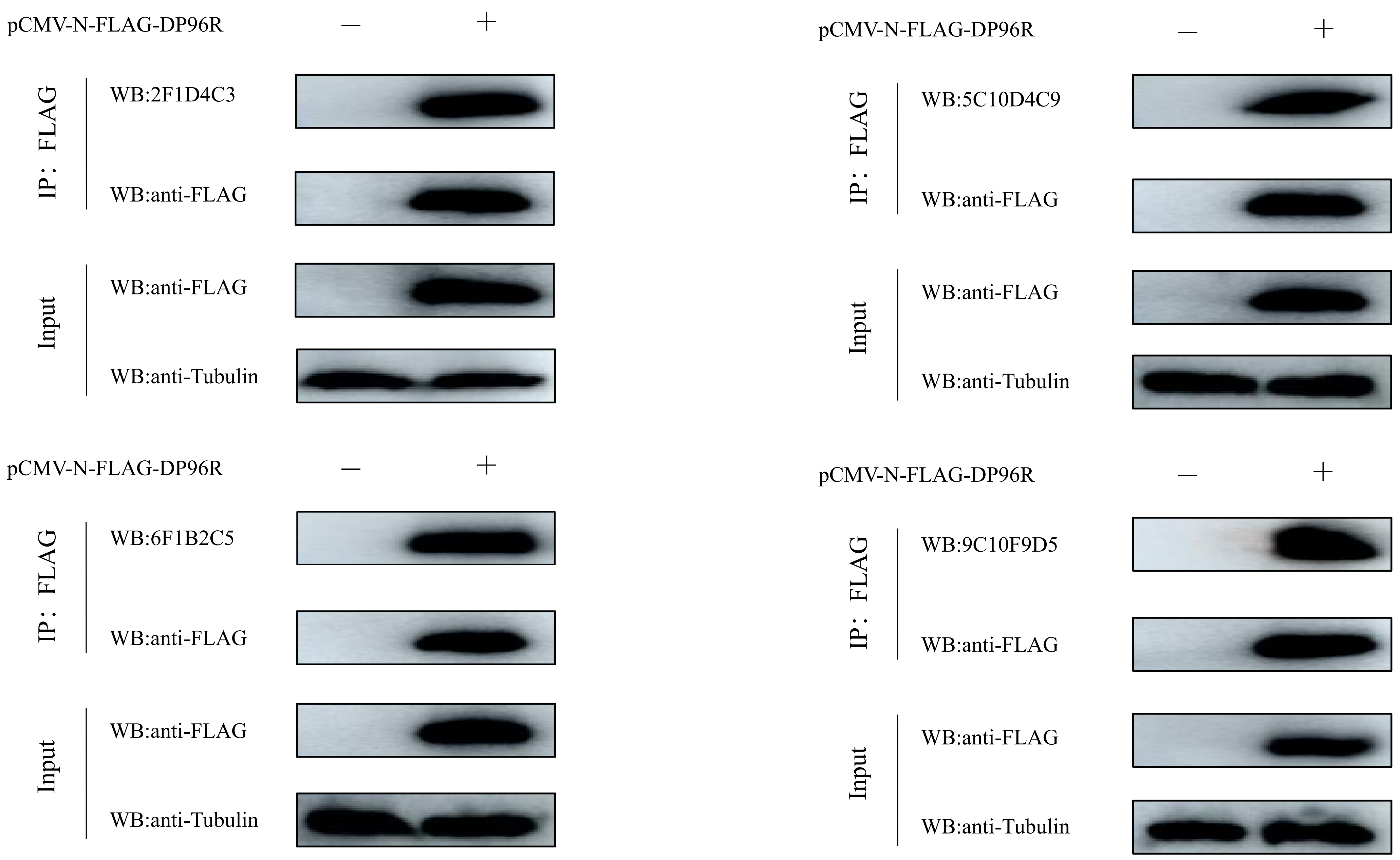
pCMV-N-FLAG-DP96R
—
+
IP：FLAG
WB:2F1D4C3
WB:anti-FLAG
Input
WB:anti-FLAG
WB:anti-Tubulin
pCMV-N-FLAG-DP96R
—
+
IP：FLAG
WB:5C10D4C9
WB:anti-FLAG
Input
WB:anti-FLAG
WB:anti-Tubulin
pCMV-N-FLAG-DP96R
—
+
IP：FLAG
WB:6F1B2C5
WB:anti-FLAG
Input
WB:anti-FLAG
WB:anti-Tubulin
pCMV-N-FLAG-DP96R
—
+
IP：FLAG
WB:9C10F9D5
WB:anti-FLAG
Input
WB:anti-FLAG
WB:anti-Tubulin

2F1D4C3

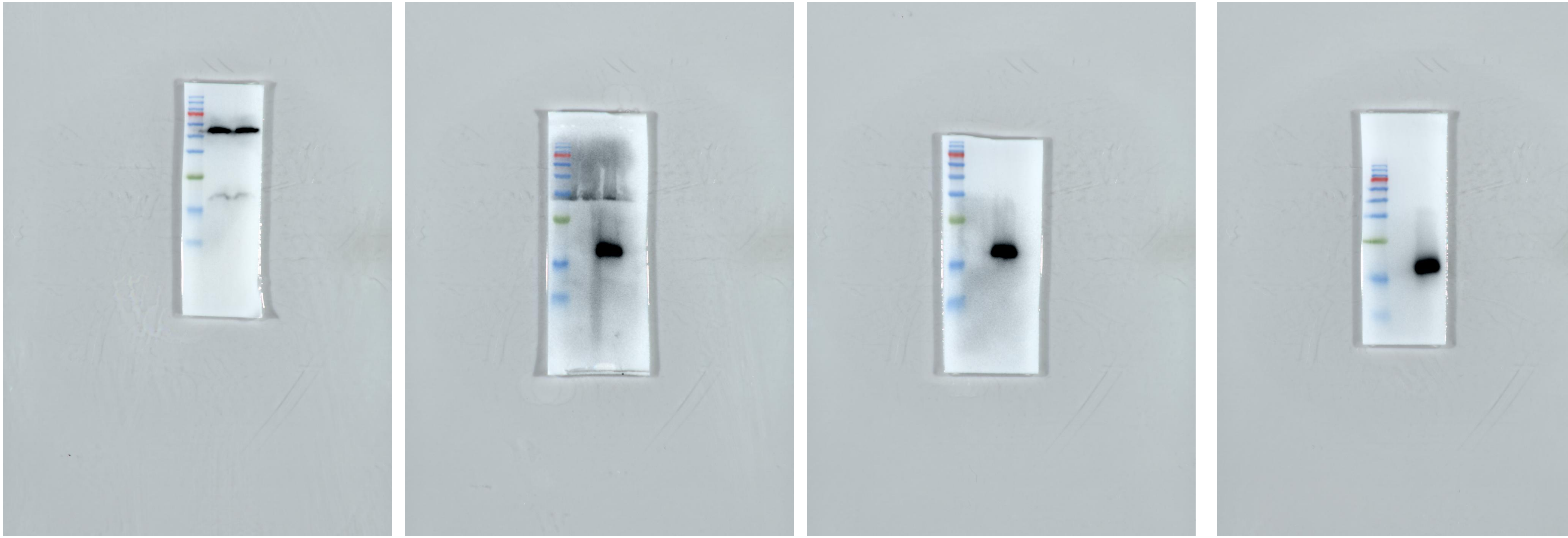

Input:anti-Tubulin Input:anti-FLAG IP:anti-FLAG IP:2F1D4C3

5C10D4C9

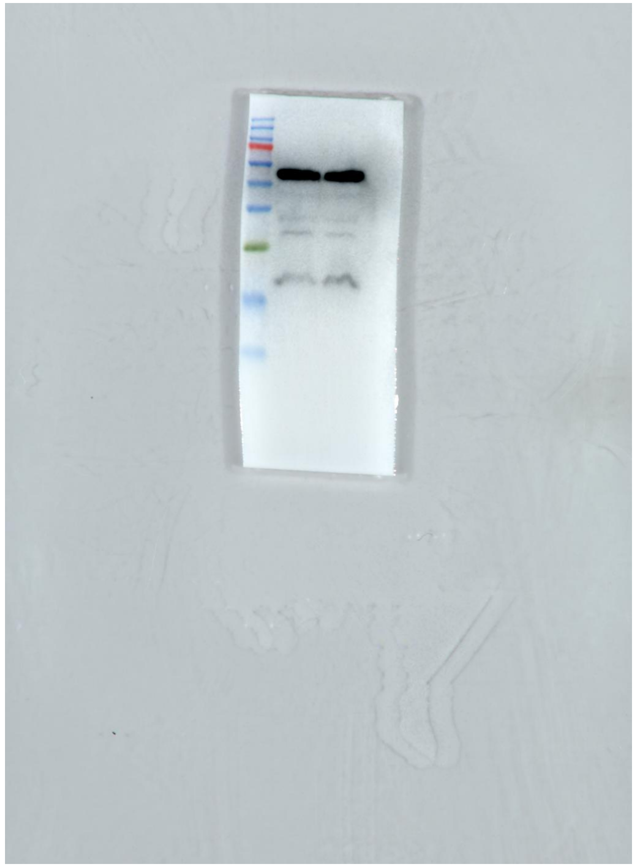

Input:anti-Tubulin

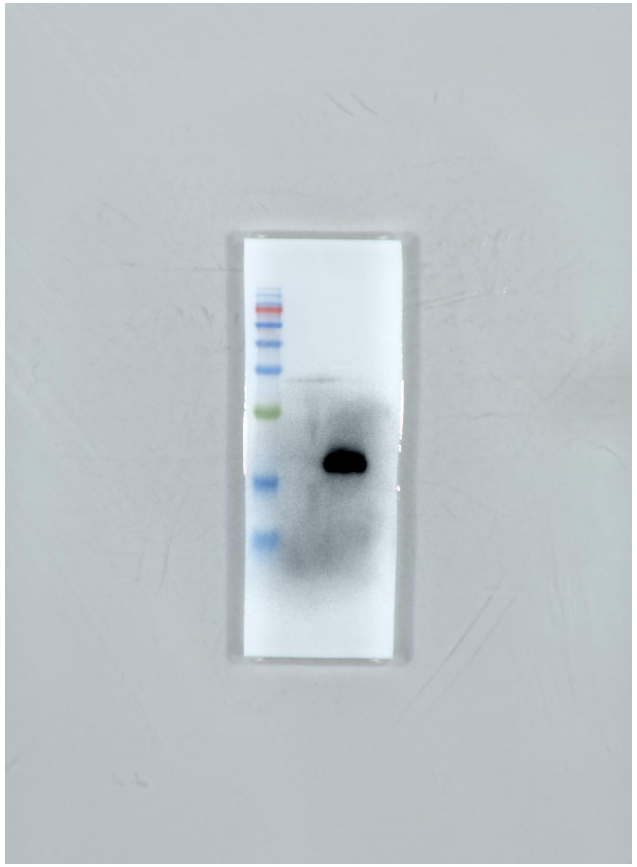

Input:anti-FLAG

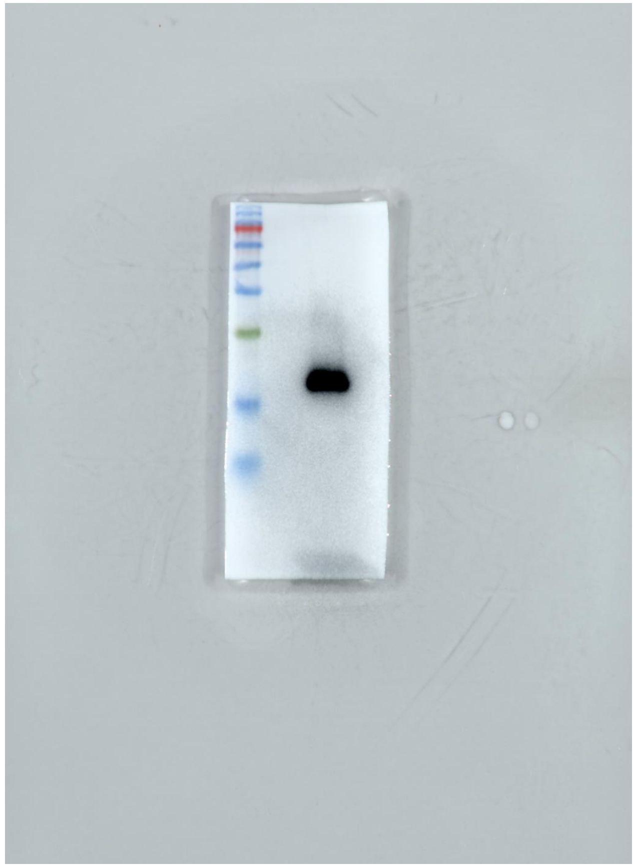

IP:anti-FLAG

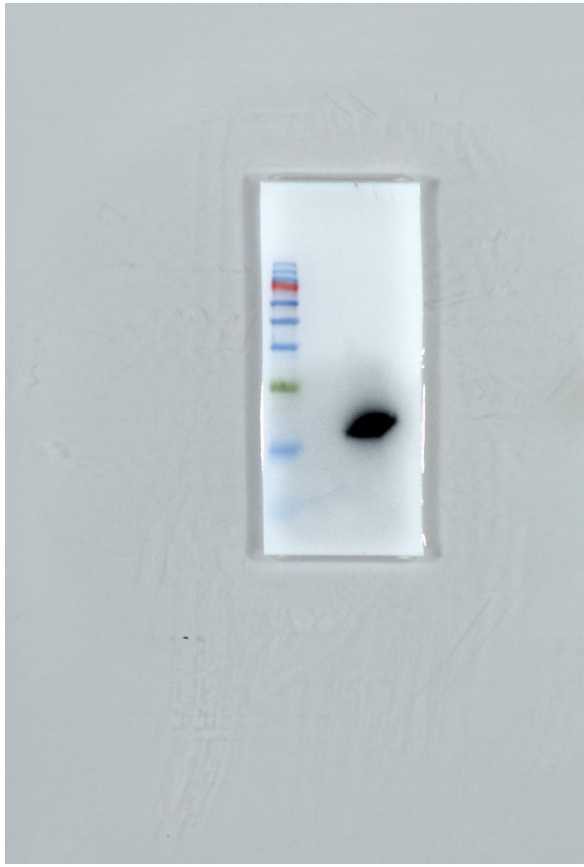

IP:5C10D4C9

6F1B2C5

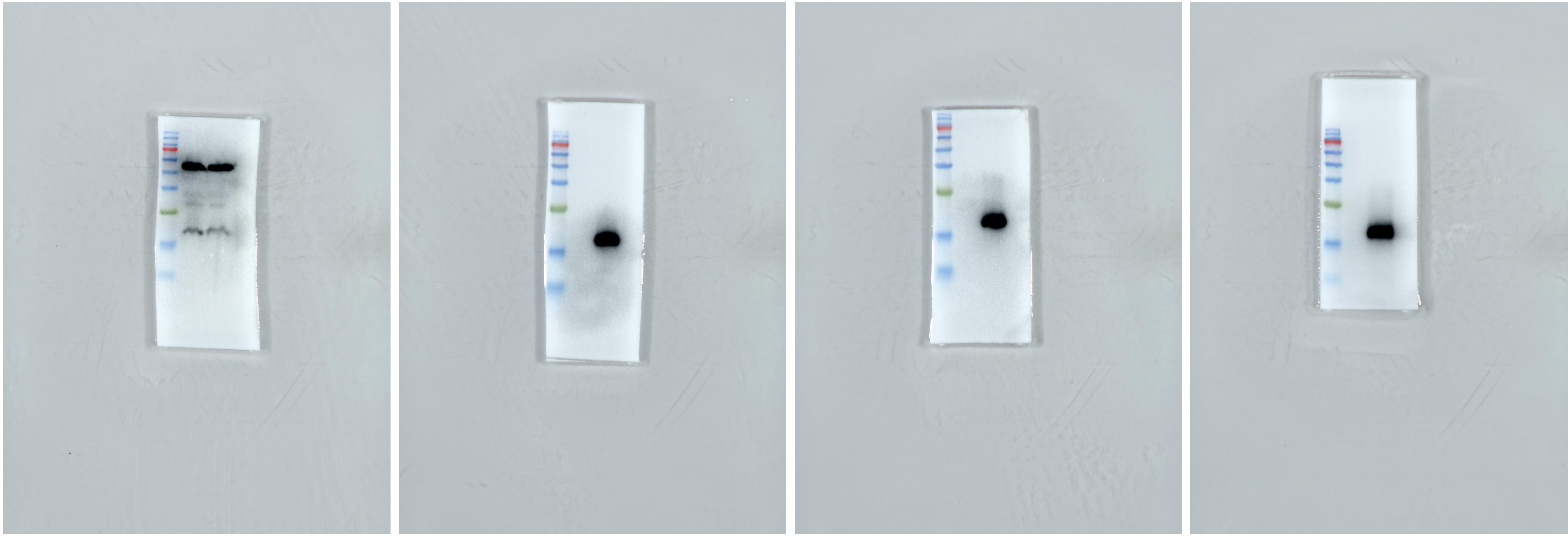

Input:anti-Tubulin Input:anti-FLAG IP:anti-FLAG IP:6F1B2C5

9C10F9D5

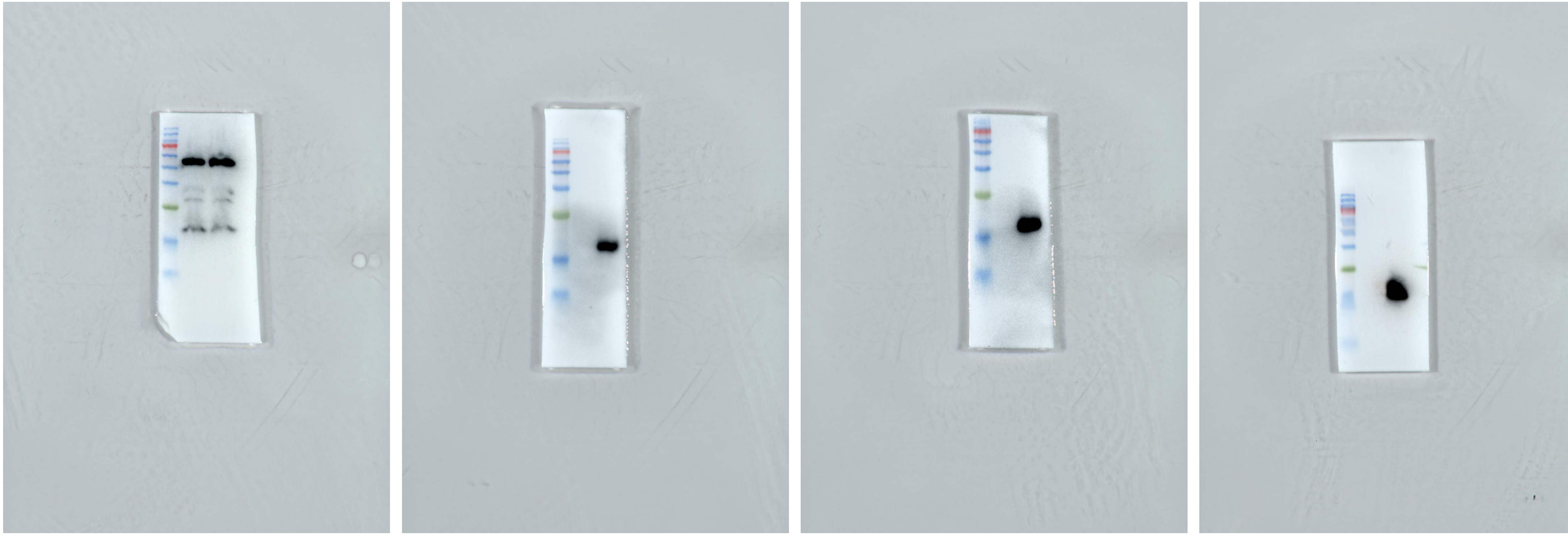

Input:anti-Tubulin Input:anti-FLAG IP:anti-FLAG IP:9C10F9D5

Fig.5

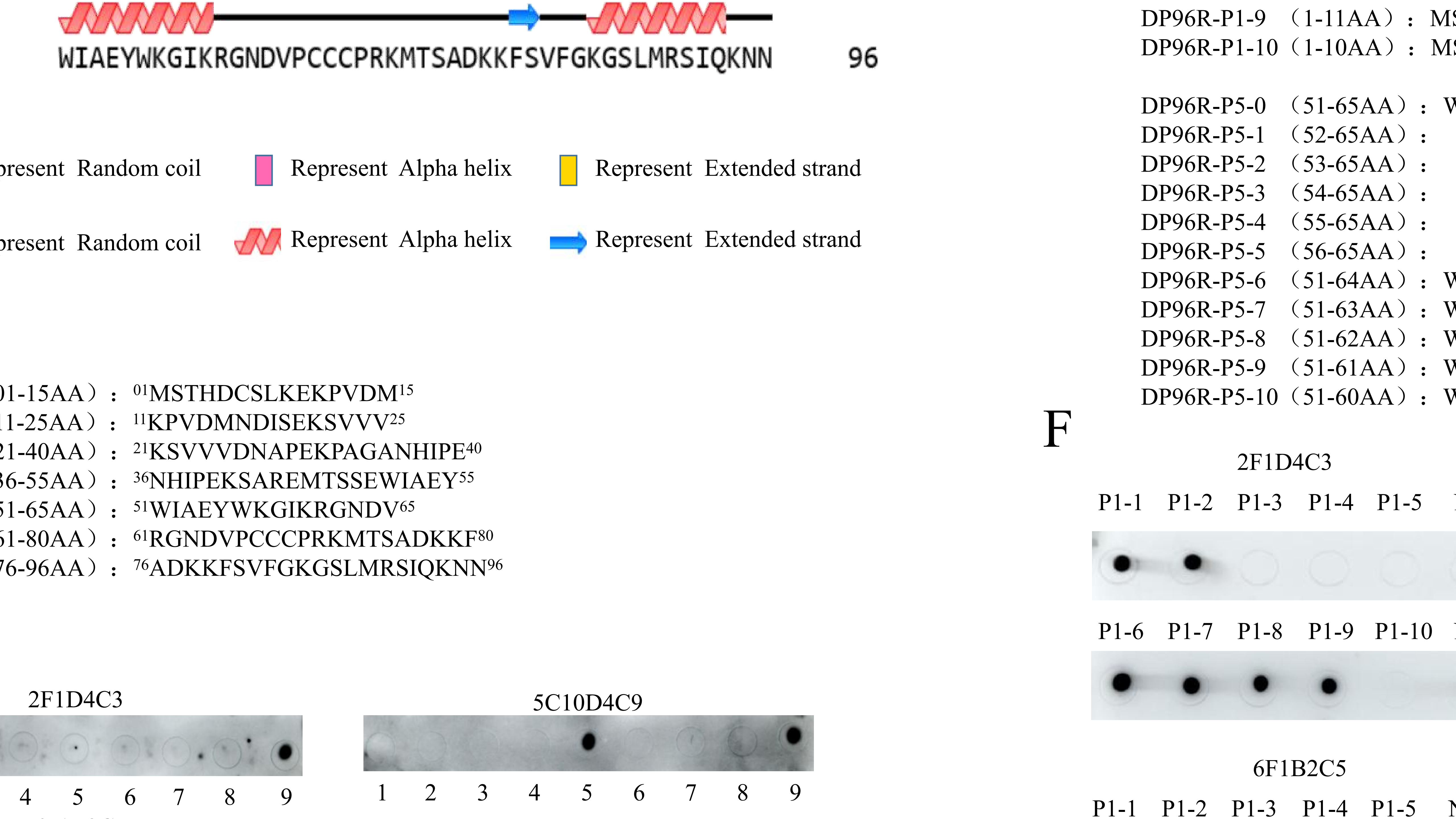
WIAEYWKGIKRGNDVPCCCPRKMTSADKKFSVFGKGSLMRSIQKNN
96
Represent Random coil
Represent Alpha helix
Represent Extended strand
Represent Random coil
Represent Alpha helix
Represent Extended strand
01-15AA) : 01MSTHDCSLKEKPVDM15
11-25AA) : 11KPVDMNDISEKSVVV25
21-40AA) : 21KSVVVDNAPEKPAGANHIPE40
36-55AA) : 36NHIPEKSAREMTSSEWIAEY55
51-65AA) : 51WIAEYWKGIKRGNDV65
61-80AA) : 61RGNDVPCCCPRKMTSADKKF80
76-96AA) : 76ADKKFSVFGKGSLMRSIQKNN96
2F1D4C3
4 5 6 7 8 9
5C10D4C9
1 2 3 4 5 6 7 8 9
DP96R-P1-9 (1-11AA) :
DP96R-P1-10 (1-10AA) :
DP96R-P5-0 (51-65AA) :
DP96R-P5-1 (52-65AA) :
DP96R-P5-2 (53-65AA) :
DP96R-P5-3 (54-65AA) :
DP96R-P5-4 (55-65AA) :
DP96R-P5-5 (56-65AA) :
DP96R-P5-6 (51-64AA) :
DP96R-P5-7 (51-63AA) :
DP96R-P5-8 (51-62AA) :
DP96R-P5-9 (51-61AA) :
DP96R-P5-10 (51-60AA) :
F
2F1D4C3
P1-1 P1-2 P1-3 P1-4 P1-5
P1-6 P1-7 P1-8 P1-9 P1-10
6F1B2C5
P1-1 P1-2 P1-3 P1-4 P1-5

2F1D4C3

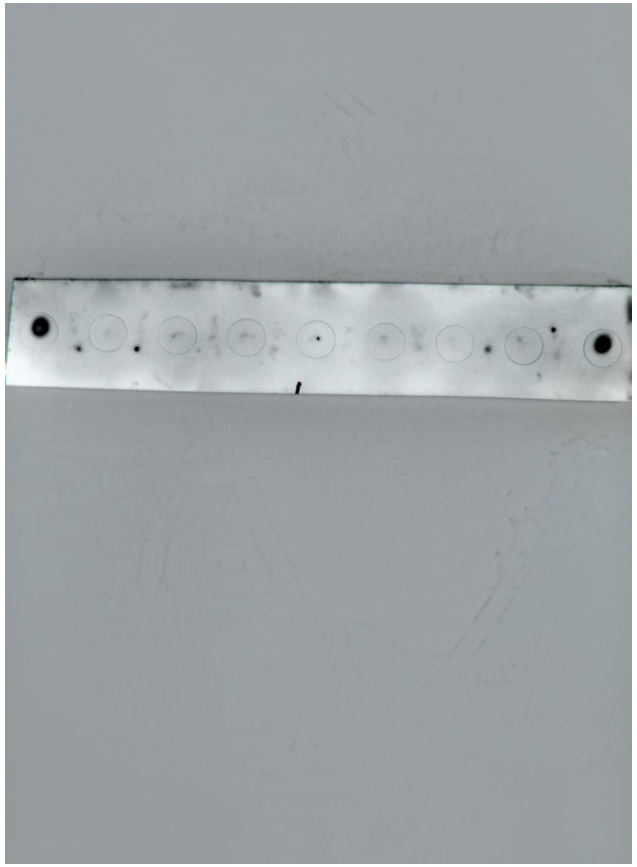

5C10D4C9

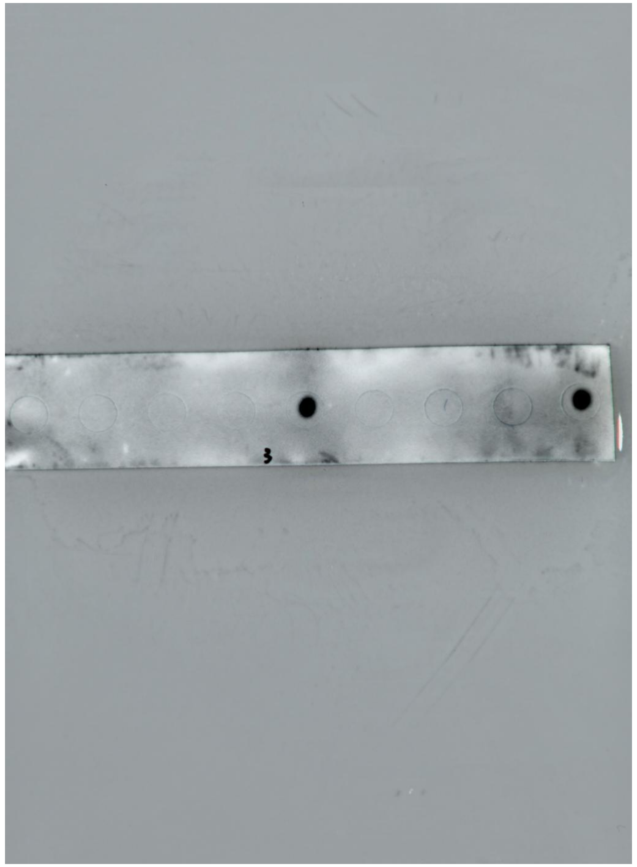

6F1B2C5

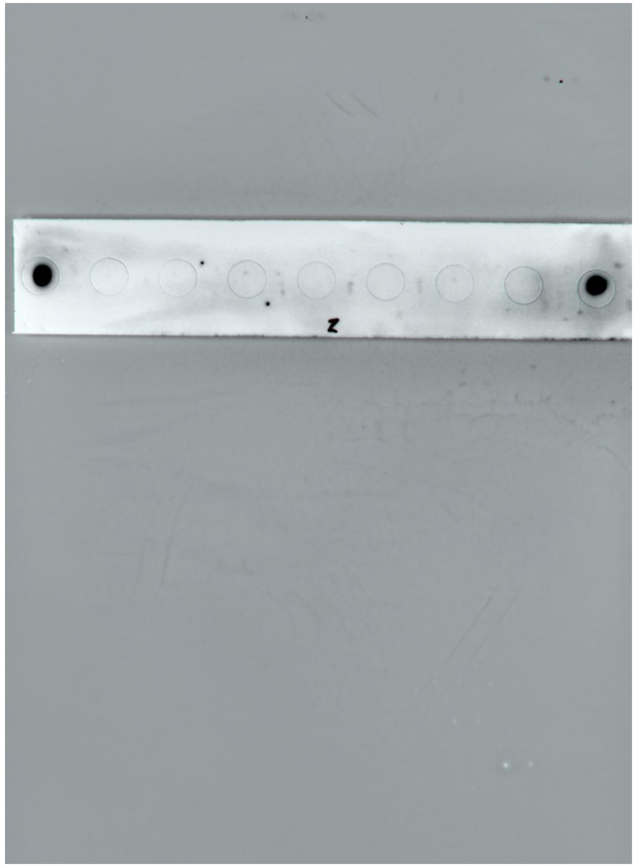

9C10F9D5

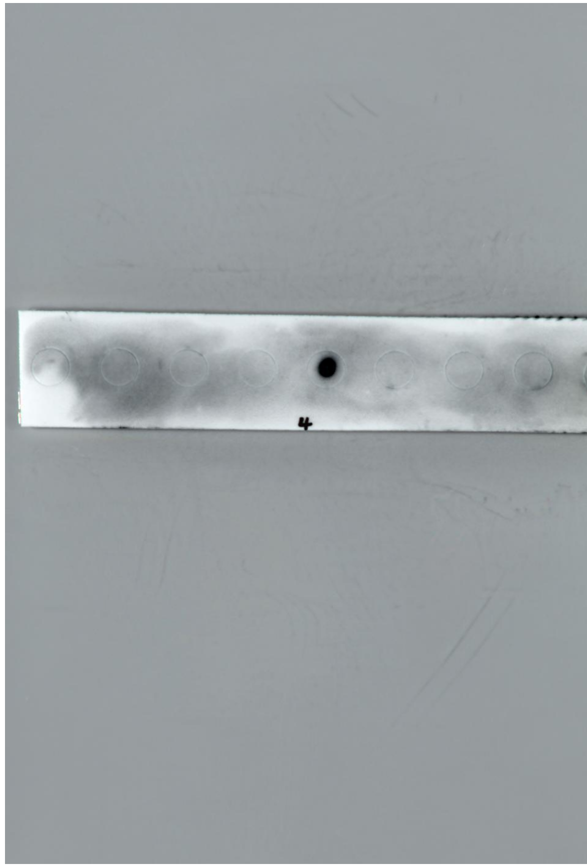

2F1D4C3 5C10D4C9 6F1B2C5 9C10F9D5

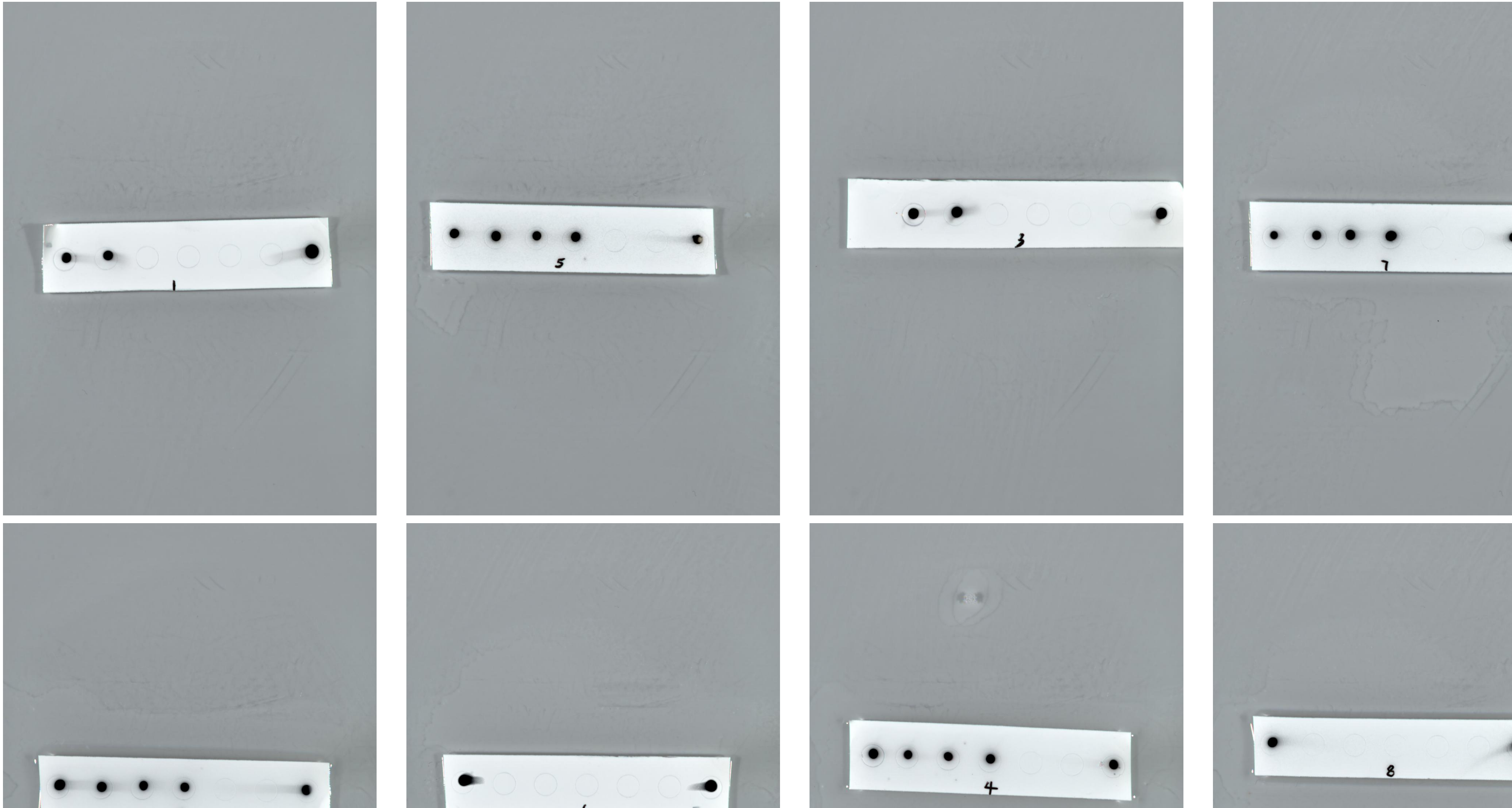

Supplement: Supplementary file 1 — Supplementary Material 1 [file 12917_2024_4043_MOESM1_ESM.pdf]
